# Role of chemokine-like factor 1 as an inflammatory marker in diseases

**DOI:** 10.3389/fimmu.2023.1085154

**Published:** 2023-02-14

**Authors:** Yutong Li, Haiyang Yu, Juan Feng

**Affiliations:** Department of Neurology, Shengjing Hospital of China Medical University, Shenyang, China

**Keywords:** chemokine-like factor 1, pathogenic mechanism, signaling pathways, immune-related disease, targeted therapy

## Abstract

Immunoinflammatory mechanisms have been incrementally found to be involved in the pathogenesis of multiple diseases, with chemokines being the main drivers of immune cell infiltration in the inflammatory response. Chemokine-like factor 1 (CKLF1), a novel chemokine, is highly expressed in the human peripheral blood leukocytes and exerts broad-spectrum chemotactic and pro-proliferative effects by activating multiple downstream signaling pathways upon binding to its functional receptors. Furthermore, the relationship between CKLF1 overexpression and various systemic diseases has been demonstrated in both *in vivo* and *in vitro* experiments. In this context, it is promising that clarifying the downstream mechanism of CKLF1 and identifying its upstream regulatory sites can yield new strategies for targeted therapeutics of immunoinflammatory diseases.

## 1 Introduction

Chemokines were discovered in the early 1970s and late 1980s (1–3). They are a group of positive-charged cytokines with molecular weights of 8-10 kDA (4, 5) that regulate the infiltration of immune cells and the release of inflammatory mediators, making them an important component of the immune system. The involvement of chemokines in disease pathogenesis relies on their binding to receptors. The selectivity of different chemokines towards receptors is closely related to their ligand types (6). In 1999, chemokines were classified into four classic families using a systematic nomenclature based on different structures: CC, CXC, C, and CX3C (7). In 2001, Han et al. discovered and reported CKLF1, a novel chemokine with atypical structure, and its three variants (8). They also identified chemokine-like factor superfamily members (CKLFSF) by reverse transcription PCR techniques in the subsequent studies (9). Owing to the presence of a MARVEL (MAL and related proteins for vesical trafficking and membrane link) domain, CKLFSF1-8 were renamed as CKLF-like MARVEL transmembrane domain-containing 1-8 (CMTM1-8) by the International Human Genetics Nomenclature Committee in 2005 (10). CMTM family (CMTMs), consisting of CKLF and CMTM1-8, is widely expressed in human tissues and plays multiple biological functions. CMTM1 negatively regulates the Ca^2+^ response in the ER and results in lymphoma cells apoptosis (11). CMTM2 is involved in the development and function of Leydig cells and modulates testicular testosterone production (12). CMTM3 mediates cell-cell adhesion and contributes to angiogenesis (13). CMTM4 plays an important role in tumors *via* regulating PD-L1 expression (14–16). CMTM5 exerts anti-atherosclerotic effects by suppressing migration and proliferation in the vessel wall (17). Deficiency of CMTM5 in oligodendrocytes leads to progressive axonopathy (18). Both CMTM5 and CMTM7 are biomarkers in human breast carcinoma (19). Targeting CMTM6 may improve the treatment of patients with clear cell renal cell carcinoma (ccRCC) (20). Over-expression of CMTM8 inhibits the invasion and metastasis of carcinoma cells, which provides a new potential target in the treatment of bladder cancer and other tumors (21, 22). CKLF1, as the most researched isoform, has potent broad-spectrum chemotactic and pro-proliferative capacity. Animal models and *in vitro* experiments have shown that CKLF1 acts in disorders affecting multiple systems by mediating different downstream signaling pathways. In this review, we outline the biology of CKLF1 and its receptors, discuss the links between CKLF1 and different diseases, and detail its downstream signaling pathways and therapies that target these mechanisms.

## 2 The structure of CKLF1 and its functions

CKLF1 is an intrinsically highly hydrophobic secretory protein obtained from pha-stimulated U937 cells. As a member of the CMTM family of proteins, CKLF1 has a contiguous CC structure similar to MDC (CCL22) and TARC (CCL17) but distinguishes from the CC family members by the absence of the C-terminal cysteine (8). There are differences in the expression levels of CKLF1 in adults and fetuses - higher levels of CKLF1 can be detected in peripheral leukocytes, spleen, lungs, and reproductive organs in adults. In contrast, higher expression was observed in fetal hearts, brains, and skeletal muscles (23). These findings suggest that CKLF1 may contribute to the physiological processes of human growth, and its abnormal expression could be a predictor of pathological states. Since CKLF1, MDC and TARC are structurally similar and are all situated on chromosome 16 (24), they may be evolutionarily conserved and have common biological activities. Furthermore, MDC and TARC bind specifically to CC chemokine receptor 4 (CCR4) and exert critical roles in allergic diseases (25). Therefore, CKLF1 may act as an immunoinflammatory factor and participate in pathogenesis through CCR4.

CCR4 is the most intensively studied CKLF1 receptor (26). It has seven typical transmembrane helices of G protein-coupled receptors (GPCRs). CCR4 is expressed in activated TH2 cells, Treg cells, activated natural killer cells, basophils, monocytes, platelets, and mature T-cell tumors (27). The C-terminal helix 8 of CCR4 facilitates the signaling and activation of chemokine receptors (28). CKLF1 has two stable secreted forms at the C-terminus – C19 peptide and C27 peptide. It can act *via* the Gi/o pathway upon binding to CCR4. C27/CCR4 exerts strong chemotactic properties, while the C19 peptide weakly activates CCR4 and may be a candidate antagonist of CKLF1 (29). As another GPCR receptor of CKLF1, CCR3 is mainly expressed in eosinophils and can also be detected on the surface of Th2 cells, basophils, and mast cells (30). Mouse model experiments have established that CCR3 plays a critical role in allergic airway inflammation (31). Moreover, the C19 peptide inhibits CCR3-mediated chemotaxis and has excellent therapeutic potential in managing allergic asthma (32). Meanwhile, CCR5 was initially identified as a receptor of chemokines, including CCL3, CCL4, and CCL5 (33). Its expression modestly reduces the risk of type 1 diabetes and celiac disease (34). CCR5+ leukocytes include Treg cells, CD4+ and CD8+ T cells, natural killer cells, dendritic cells, monocytes, and macrophages (35). A study by Chen et al. reported that CCR5 mediates neutrophil migration and participates in cerebral ischemia/reperfusion (I/R) injury by acting as a receptor for CKLF1 (36). Given the structural similarity of CKLF1 to members of the CC chemokine family, there are likely to be other typical or atypical CKLF1-binding GPCRs. Therefore, a further expansion of the CKLF1 receptor family is to be anticipated.

CKLF1 interacts with various cells, including neutrophils, lymphocytes, monocytes, and neuronal cells, playing an integral role in the transport of immune cells and the production of immune mediators. The activated GPCRs cause the G protein subunits to dissociate and the GDP to exchange with GTP following the binding of CKLF1 to GPCRs as CCR4, CCR3 and CCR5. The Gα and Gβ subunits further activate downstream signaling pathways and exert broad chemotactic activities. Dendritic cells (DCs) possess potent antigen-presenting functionality and are involved in Th1/Th2 polarization (37). *In vitro* experiments suggest CKLF1 may stimulate DC maturation through the NF-κB and MAPK (mitogen-activated protein kinase) pathway (38, 39). It has been found that peptides C19 and C27 stimulated the secretion of IL-12 in DCs and promoted the production of IFN-γ, which in turn affected the ability of DCs to activate Th1 cells while not affecting the activation of Th2 cells. The expression of CCR4 was detected on DCs, but whether the mentioned processes were mediated *via* CCR4 needs further investigation (40). In addition to participating in DC activation, CKLF1 may also regulate the activation of T lymphocytes. It has been shown that the mRNA levels and protein expression levels of CKLF1 were increased in activated CD4+ and CD8+ lymphocytes in a time-dependent manner (41). CD4+ T cells expressing CXCL5 and CD57 are localized explicitly to germinal centers and are called GC-Th cells. GC-Th cells are activated in a CD28 co-stimulatory signal-dependent manner, producing and secreting large amounts of CXCL13 as critical chemokines for B-cell entry into lymphoid follicles. Gene expression profiling revealed that GC-Th cells might induce the expression of CKLF1 and participate in the above process, but the exact regulatory mechanism is not yet clear (42). In addition, CKLF1 also mediates an important non-immune function, namely pro-proliferative capacity, targeting skeletal muscle cells, vascular smooth muscle cells, and bone marrow cells, providing therapeutic orientations for myasthenia gravis, vascular diseases, and hematological disorders (43–45).

## 3 CKLF1-related diseases

Various chemokines are involved in the development of many acute and chronic diseases by regulating the inflammatory environment. Chemokines do not correspond to diseases in a piecewise manner but are intertwined into networks. Many chemokine-receptor combinations may exert similar cellular functions in different diseases, while the same chemokine may bridge specific signaling pathways with different effects on disease progression. The role of CKLF1 in coordinating immune responses in different systemic diseases has been reported, and it would be meaningful to study the effects of CKLF1 targeting different cells in these diseases. In this section, we will present diseases associated with the undesirable effects of CKLF1, focusing on the underlying mechanisms of CKLF1 actions in different systemic diseases and the evidence supporting these effects of CKLF1. Here we list references containing reviews related to the diseases explored for readers to follow in more detail (**Table 1**).

**Table 1:** The association of CKLF1 with diseases.

CKLF1-mediated diseases	Main targeting cells	Effect on disease	Related Mechanisms	References
Cerebral ischemia	Neutrophils; Microglia	Early cerebral ischemia: promotes neutrophil migration, microglia M1 polarization, BBB destruction, and aggravates cerebral ischemic injury. Late cerebral ischemia: recruits nerve cells and promotes vascular regeneration for neurological recovery.	AKT/GSK-3β pathway; MAPK pathway; NF-κB pathway	(36, 46–51)
Brain development	SH-SY5Y cells and cortical neurons	Induces neuronal migration and promotes brain development	Non-extracellular calcium-dependent tyrosine kinase pathway	(52–54)
Bronchial asthma	Th2 lymphocytes and eosinophils	Recruits inflammatory cells to the bronchial mucosa, promotes the proliferation of bronchial smooth muscle cells and fibroblasts, and aggravates pulmonary fibrosis in bronchial asthma	NF-κB pathway	(55–59)
Allergic rhinitis	Th2 lymphocytes and eosinophils	Induces migration of inflammatory cells, promotes IgE production and the release of inflammatory factors and aggravates allergy symptoms	Not yet mentioned	(32)
Arthritis	Synovial lining cells Leukocytes	Causes proliferation of synovial lining cells, vascular proliferation and fibrosis, and diffuse inflammatory cell infiltration, which are involved in the mechanism of arthritis	MAPK pathway; NF-κB pathway	(60–63)
Psoriasis	Lymphocytes Endothelial cells	Induces lymphocyte migration to the skin, promotes microvascular dilation and endothelial cell proliferation and mediates the development of psoriasis	MAPK pathway	(64, 65)
Lupus Nephritis	Leukocytes	Promotes the accumulation of inflammatory cells to the site of injury where immune complexes are deposited and thereby aggravates the injury	Not yet mentioned	(66)
Antiphospholipid syndrome	Blood platelets	Affects platelet activity and function; is involved in hemostasis and thrombosis	Not yet mentioned	(67)
Inflammatory myopathy	Lymphocytes Muscle fibers	Attracts lymphocytes with regenerating muscle fibers to the site of inflammation and participates in the development of polymyositis (PM) and dermatomyositis (DM)	Not yet mentioned	(68)
Atherosclerosis and RS	Vascular smooth muscle cells (VSMCs) Mononuclear cells	Induces monocyte adhesion to vascular endothelium, promotes vascular smooth muscle cell migration, and accelerates thrombosis.	PI3K/AKT/NF-κB pathway	(69–71)
Abdominal Aortic Aneurysm	Macrophages Lymphocytes	Upregulates MMP-2 expression and accelerates (aortic wall structural protein) extracellular matrix degradation, leading to the development of AAA.	Not yet mentioned	(72)
Keloid scars	Not yet mentioned	Not yet mentioned	Not yet mentioned	(73)
Hepatocellular carcinoma	Tumor cells	Inhibits apoptosis, promotes malignant transformation, and induces HCC development and metastasis	IL6/STAT3 signaling pathway	(74)

### 3.1 CKLF1 and neurological diseases

#### 3.1.1 Cerebral ischemia

Ischemic stroke is one of the leading causes of mortality and disability worldwide, resulting in 6 million deaths and 5 million permanent disabilities yearly (75). Although reperfusion may help to rescue the ischemic semidark zone, it can exacerbate neuroinflammation and worsen brain damage. Blood-brain barrier (BBB) disruption induced by neutrophil migration and release of pro-inflammatory factors participates in the pathophysiological process of cerebral ischemia-reperfusion (I/R) injury. Following the onset of stroke, activated microglia and astrocytes secrete pro-inflammatory mediators such as chemokines, with neutrophils migrating to the lesion site within a few hours. The infiltrating neutrophils release cytokines, including proteases, which activate glial cells *via* positive feedback, synergistically destroying the BBB and causing neuronal necrosis (76). The neutrophil/lymphocyte ratio is of great value in estimating the severity and prognosis of cerebral ischemic injury (46). Microglia are the primary cells governing the intrinsic immune response of the brain. Microglia/macrophage polarization has been proven to regulate the development of various central nervous system disorders, including stroke, multiple sclerosis, and spinal cord injury (77, 78). Microglia are activated into two types in early cerebral ischemia: the pro-inflammatory M1 microglia, and the anti-inflammatory M2 microglia. The brain microenvironment favors M1 polarization, leading to brain injury progression and neurological deficits.

Preliminary experimental studies have confirmed that CKLF1 plays a central role in I/R injury. Research using a rat model of cerebral ischemic injury showed that CKLF1 expression was significantly elevated at the injury site after 3 hours and peaked after 48 hours. Knockdown of CKLF1 by HIF-1α-guided AAV in the ischemic area of the rat brain reduced the size and water content of the infarct area, confirming that CKLF1 exerts a pro-inflammatory effect in the early stage of cerebral ischemia to aggravate the injury. Immunohistochemical staining and an MPO activity assay showed that CKLF1/CCR5 mediated neutrophil migration through the AKT/GSK-3β pathway (75). In contrast, an earlier study reported that an anti-CKLF1 antibody inhibited neutrophil infiltration *via* the MAPK pathway (47). The different results may originate from the differences in CKLF1 distribution in the brain. The expression of CKLF1 is spatially specific in cerebral ischemic injury and primarily occurs in the cortex, thalamus, and hippocampus (46). Therefore, selective knockdown of CKLF1 at different sites may affect the experimental results differently. In addition, the selection of the time window may bring limitations to the experimental results - CKLF1 may mediate different signaling pathways or combine multiple signaling pathways to effect at different stages after the onset of ischemia-reperfusion injury. *In vitro* experiments have demonstrated that CKLF1 partially depends on CCR4 to regulate M1 polarization of BV2 microglia and induce oxygen-glucose deprivation/reperfusion (OGD/R) injury (48). Experiments using a mouse MCAO (middle cerebral artery occlusion) model showed that either exogenous or endogenous CKLF1 could promote M1 polarization in microglia during early cerebral ischemia, and the polarization process was associated with CKLF1/CCR4 axis-mediated activation of the NF-κB pathway (49). AQP4, MMP-9, and tight junction (TJ) proteins are markers that reflect BBB function. Overexpression of AQP4 is a significant cause of brain water imbalance (50). Inhibition of CKLF1 reduced AQP4 and MMP-9 levels, upregulated the expression of TJ proteins, including ZO-1 and Occludin, and attenuated brain edema in rats (51).

#### 3.1.2 Brain development

The development of the cerebral cortex is accomplished through complex modulations on neurogenesis, neuronal migration, and neuronal connectivity. Disruptions in these processes can lead to neuropsychiatric disorders such as drug-resistant epilepsy, intellectual disability, and schizophrenia (79). Chemokines play a role in brain development by regulating synaptic transmission, cell migration, and other processes through autocrine or paracrine signaling (80). Not only is CXCR4/CXCL12 involved in inducing neurogenesis (81), but the CXCR4/CXCR7/CXCL12 axis may also guide cortical neuronal migration (82). *In vitro* experiments have shown that stromal cell-derived factor-1α (SDF-1α) acted on the migration of various neurons in the dentate gyrus, cerebral cortex, and brainstem nuclei. RANTES was found to have a stimulating effect on the migration of dorsal root ganglion cells (83). The expression level of CKLF1 mRNA in the adult brain was found to be well below that of the fetal brain (52). This discrepancy in expression may indicate that CKLF1 is involved in brain developmental processes. It was observed that CKLF1 induced the migration of neurons in the rat cerebral cortex in a dose-dependent manner at concentrations of 200 nM and 2000 nM (53), and this process was mediated through the non-extracellular Ca^+^-dependent tyrosine kinase pathway (54).

### 3.2 CKLF1 and respiratory diseases

#### 3.2.1 Bronchial asthma

Bronchial asthma is a heterogeneous chronic inflammatory disease with inflammation, hyperresponsiveness (AHR), and remodeling of the airways as the primary pathophysiological mechanisms characterized by Th2 lymphocyte and eosinophil infiltration. Airway epithelial cells and alveolar macrophages generate various chemokines in response to allergic or non-allergic stimuli, recruiting inflammatory cells toward the bronchial mucosa and participating in the onset and progression of asthma. The role of several chemokine receptors in asthma has been reported - CCR4 was found to be highly expressed in Th2 cells, and CCL17/CCR4, CCL22/CCR4 mediated the migration of Th2 cells and triggered allergic airway responses (84–86). CCR4-targeting inhibitors such as thymus and activation-regulated chemokine (TARC-PE38) have alleviated airway inflammation (87). Meanwhile, CCR3 is involved in eosinophil transport as a major receptor and acts with CCR4, CCR5, CCR6, CCR8, and CXCR4 to induce airway inflammation and AHR (88).

Mouse models provide excellent insights into the roles of specific chemokines in the respiratory system. Intramuscular injection of CKLF1 plasmid to BALB/c mice increased the total leukocyte count and eosinophil percentage in BALF, causing pathological lung modifications consistent with bronchial asthma (55). This process is facilitated by the CKLF1/CCR4-mediated NF-κB pathway (56). C19 peptide inhibited Th2 cell responses, eosinophilia, and AHR in a mouse allergic asthma model by acting on CCR4 and CCR3. In contrast, the C27 peptide caused a mild increase in total leukocytes in BALF of asthmatic mice, which was not statistically significant (57). It was found that inhibition of CKLF1 binding to CCR4 with antagonists alleviated airway symptoms in asthmatic mice and attenuated airway remodeling and lung fibrosis caused by epithelial cell and fibroblast proliferation (58). A clinical study confirmed that CKLF1 mRNA expression was significantly higher in the peripheral blood of asthmatic patients compared to controls and that CKLF1 level in the airways of these patients was much higher than in normal subjects (59).

#### 3.2.2 Allergic rhinitis

Allergic rhinitis is an inflammatory disease of the nasal mucosa prominently marked by elevated IgE. It has pathogenesis similar to that of bronchial asthma featuring typical biphasic responses - with the early phase characterized by IgE activating basophils and mast cells to secrete cytokines, causing allergic symptoms including running nose and itching; then the pro-inflammatory factors further activate inflammatory cells, including eosinophils, initiating late-phase responses that lead to sustained symptoms (89). Th2 cells promote the synthesis and secretion of IgE, which is critical in the above responses (90). Intranasal and intraperitoneal administration of C19 peptide to allergic rhinitis mice relieved symptoms and decreased serum IgE concentrations, with better efficacy of intranasal than intraperitoneal delivery (32). These findings substantiate that C19 peptide may be used as a therapeutic agent for allergic rhinitis by intranasal administration.

### 3.3 CKLF1 and rheumatic diseases

#### 3.3.1 Arthritis

The main types of arthritis include rheumatoid arthritis (RA), osteoarthritis (OA), and ankylosing spondylitis (AS), with characteristic pathological changes being infiltration of the periarticular synovial tissue by mixed inflammatory cells. The successful development of TNF antibodies and soluble TNF receptor antagonists confirms the vital contribution of TNF in these diseases. TNF is known to induce the production of various chemokines, regulate inflammatory cell infiltration and vascular proliferation, and participate in the pathogenesis of arthritis. Several studies have identified receptors for chemokines, including CCR2, CXCR2, and CXCR3, on infiltrating cells from patients with arthritis (91–93). Among them, the presence of CCR5 is often associated with the deficiency of rheumatoid factor (94).

Given the scarcity of animal models of arthritis concerning CKLF1, most evidence was derived from clinical studies. A study (60) included 16 patients with OA, 15 with RA, and 10 with AS and measured levels of CKLF1, CCR4 mRNA, and plasma inflammatory markers. The results showed that CKLF1 levels were increased in synovial membranes of patients with RA, OA, or AS, and patients with RA presented with concomitant upregulation of CCR4 mRNA expression. The authors theorized that CKLF1 in RA acts through binding to CCR4, while in OA and AS, it may mediate disease progression through pathways other than the CKLF1/CCR4 axis. Several other clinical studies have found a positive correlation between CKLF1 levels and C-reactive protein (CRP)/sedimentation (ESR) in patients with RA, suggesting that CKLF1 may be a sensitive indicator for evaluating disease activity. However, in patients with OA and AS, findings contradict previous reports, which require verification through further studies (61–63).

#### 3.3.2 Other rheumatic diseases

In addition to arthritis, CKLF1 also finds a role in other rheumatic diseases. CKLF1 and CCR4 levels were significantly elevated at the lesion sites in patients with psoriasis (64). The proposed mechanism is that CKLF1 induces lymphocyte migration to the skin and promotes microvascular dilation and endothelial cell proliferation, mediating the pathogenesis of psoriasis. The role of the C19 peptide in psoriasis is still controversial. It has been shown that the C19 peptide significantly reduced the number of neutrophils in patients with psoriasis and exerted a protective role in psoriasis through the MAPK signaling pathway (65). Despite being weaker than the C27 peptide, the C19 peptide has also been shown to promote the proliferation of vascular endothelial cells (64). The different biological effects induced by the C19 peptide may result from the variability of the local inflammatory environment (95). CKLF1 is expressed at low levels in normal kidneys and exerts a physiological chemotactic function. Overexpression of CKLF1 resulted in increased urinary protein in mice, which exhibited the pathological modifications of lupus nephritis (LN). CKLF1 levels in LN patients were positively correlated with the lupus activity index and could be used as a valid predictor of disease activity (66). Antiphospholipid syndrome (APS) manifests itself primarily by thrombosis and recurrent obstetric events. CKLF1 may compromise platelet activity and function by participating in the hemostatic and thrombotic processes, leading to the aggravation of APS (67). In inflammatory myopathies, intratubular thrombin induces the expression of CKLF1. CKLF1 may be a marker of myofiber regeneration in its ability to attract lymphocytes and regenerate myofibers (68).

### 3.4 CKLF1 and circulatory diseases

Atherosclerosis is known as an inflammatory mechanism-mediated disease. Studies on chemokines in atherosclerosis models are relatively well established. In response to inflammatory injury, the release of chemokines from activated endothelial cells and arterial smooth muscle cells induces monocyte adhesion to the vascular endothelium and contributes to forming foam cells. In addition, chemokines promote the migration of smooth muscle cells to the endothelium and their attachment to the plaque to form a thrombus (96). Monocyte chemotactic protein 1 (MCP-1), Fractalkine (CX3CL1), RANTES (CCL5), and eotaxin (CCL11) are involved in the development of atherosclerosis (97–100). Restenosis (RS) is highly prevalent at six months after percutaneous transluminal coronary angioplasty, with restenosis rates of up to 30-50% (101) in patients treated with balloon angioplasty or bare metal stents and still up to 12-20% with drug-eluting stents (102). The pathogenesis of RS is very similar to that of atherosclerosis, with extensive involvement of chemokines.

Many researchers have applied a balloon injury rat model to explore the effects of CKLF1. Early studies found that CKLF1 levels were significantly elevated in the neointima after injury and co-localized with vascular smooth muscle cells (VSMCs). C19 peptide potently inhibited VSMC migration and endothelial proliferation after CKLF1 transfection (69). A subsequent study showed that CKLF1 acted on G2/M-phase VSMCs to regulate the balance between proliferation and apoptosis and accelerate neoplastic endothelial formation. This process was regulated through the PI3K/AKT/NF-κB pathway (70). These results were confirmed in later clinical studies. It was found that the expression of CKLF1 and vascular adhesion molecule-1 (VCAM-1) was significantly increased in human carotid plaques compared to controls. Meanwhile, CKLF1 promoted the aggregation of human aortic smooth muscle cells (HASMCs) and monocyte adhesion through the NF-κB/VCAM-1 pathway (71).

### 3.5 CKLF1 and tumors

The role of chemokines in tumors is not limited to the recruitment of leukocytes but may also interfere with cell proliferation and the generation of new blood vessels. Melanoma growth-stimulating activity (MGSA) and IL-8 may act as tumor growth factors that directly contribute to the development of melanoma and non-small cell lung cancer (103, 104). It was revealed that CXC chemokines containing the Glu-Leu-Arg motif (ELR motif) promoted angiogenesis, while non-ELR CXC chemokines counteracted it (105). This may indicate that the bidirectional regulatory effects of chemokines in tumors are structure-related. As a novel chemokine, the structure of CKLF1 is highly characteristic. Exploring the role of CKLF1 in tumors may provide more possibilities for targeted therapy.

Abdominal aortic aneurysm (AAA) is closely correlated with structural protein degradation of the aortic wall mediated by inflammatory mechanisms. A rat AAA model study showed that CKLF1 levels were elevated in the AAA group compared to controls, with a significant positive correlation with MMP-2 levels, confirming that CKLF1 promotes AAA through upregulating the expression of MMP in the extracellular matrix (72). It was also found that basal CKLF1 levels were higher in keloid individuals than in those without keloids, and the expression of CKLF1 mRNA was higher in keloid tissues than in normal tissues, suggesting that CKLF1 could be instrumental in predicting keloids. However, the exact mechanism remains to be further elucidated (73). Most clinical studies on CKLF1 in malignancies have focused on hepatocellular carcinoma. One study reported that in patients with hepatocellular carcinoma, CKLF1 levels were significantly higher in cancer tissues compared to normal tissues, and the expression levels were higher in advanced cancer than in the early stages. Additionally, this research demonstrates that CKLF1 mediated its oncogenic effects through the IL-6/STAT3 signaling pathway and could assist in the staging and prognostic assessment of patients with hepatocellular carcinoma (74).

## 4 Mechanisms of CKLF1-mediated pathogenesis

The activation and crosstalk of diverse intracellular signaling pathways are involved in the mechanism of CKLF1-mediated pathologies, including the NF-κB pathway, MAPK pathway, JAK/STAT3 pathway, and PI3K/AKT pathway.

### 4.1 NF-κB signaling pathway

NF-κB is a crucial nuclear transcription factor widely present in animal tissues and participates in the regulation of processes, including immune inflammatory responses, cell proliferation and differentiation. The NF-κB transcription factor family consists of five proteins that share the Rel homologous structural domain: RelA (p65), RelB, cRel, NF-κB1 (p105/p50), and NF-κB2 (p100/p52). Without stimulatory signals, NF-κB is complexed with IκB in the cytoplasm and remains in dynamic homeostasis. In contrast, when the NF-κB pathway is activated by stimulating factors, it exerts effects through classical and non-classical pathways (alternative pathways) (106). In the classical pathway, PRRs (pattern recognition receptors), inflammatory cytokine receptors, TCRs (T cell receptors), and BCRs (B cell receptors) act as stimulatory signals to the IκB kinase (IKK) complex, causing IκB phosphorylation, which leads to the release of NF-κB dimers and their translocation to the nucleus, promoting the transcription of a variety of target genes (107). The NF-κB alternative pathway can be activated by members of the TNFR superfamily, with p100 processing being a key link in this pathway. An NF-κB-inducing kinase (NIK) and IKKα act as essential factors for p100/RelB processing, which leads to the activation of P52/RelB. This heterodimer may bind to different promoter regions and exert diverse biological effects upon entry into the nucleus (108).

**Figure 1:**
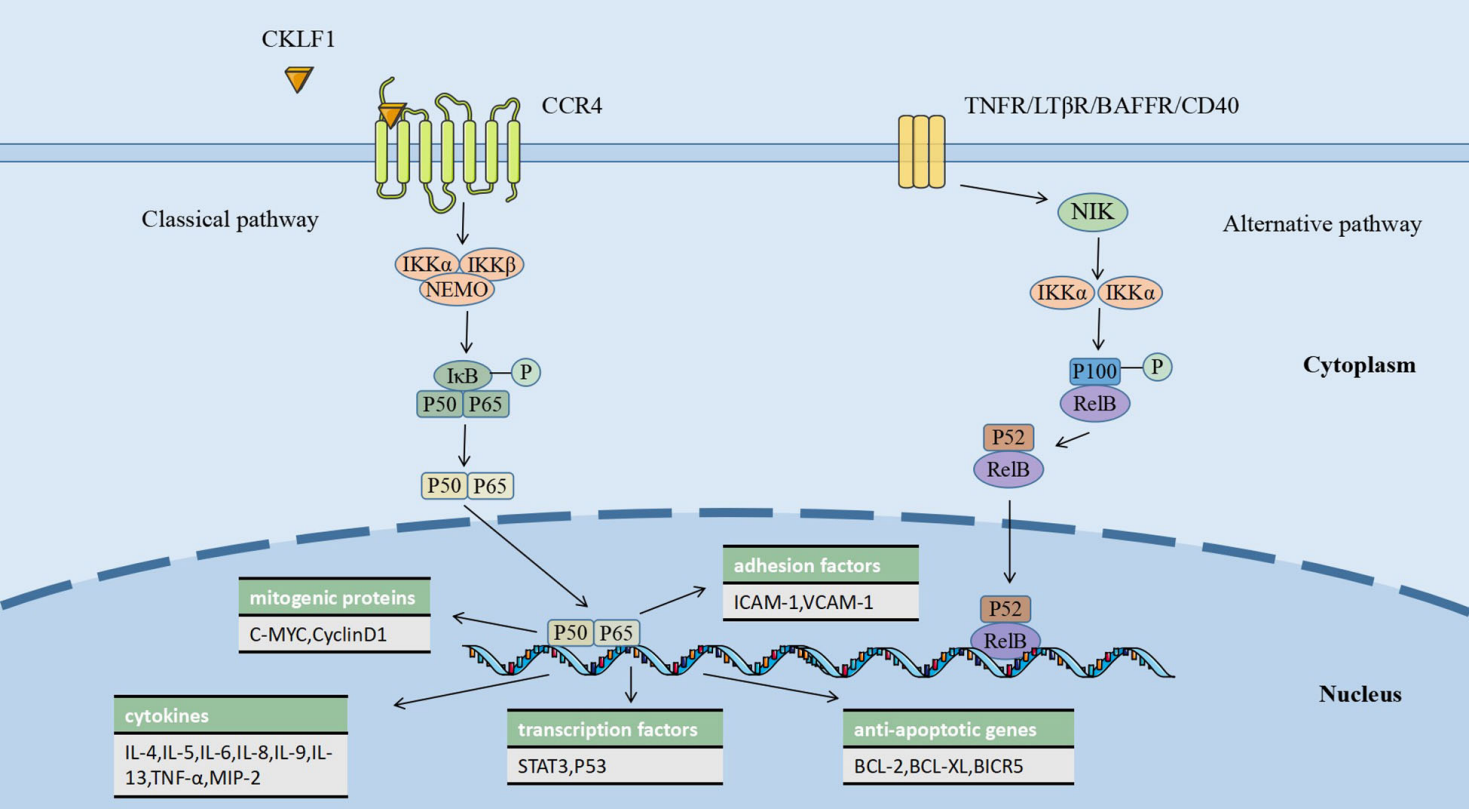
CKLF1 binds to CCR4 and activates the NF-κB classical pathway, causing a variety of biological processes, including the release of inflammatory factors in the nucleus, the transcription of anti-apoptotic genes, and the expression of mitogenic proteins, which are involved in the regulation of immune-inflammatory responses.

Unlike other ligands of CCR4, such as TRAC and MDC, CKLF1 can activate the NF-κB classical signaling pathway involved in disease-causing mechanisms, which may have to do with its specific structure. A study on human amniotic mesenchymal stromal cells showed that CKLF1 mediates monocyte adhesion and smooth muscle cell (SMC) migration through the NF-κB/VCAM-1 pathway and that the use of PDTC, an NF-κB inhibitor, suppressed the CKLF1-induced elevation of VCAM-1 (71). A mouse MCAO model study showed that CKLF1 binding to CCR4 activated the NF-κB pathway and promoted microglia/macrophage polarization toward the M1 phenotype (49). CKLF1 also induces significant airway inflammation and pathological changes in mouse lungs by triggering NF-κB-regulated gene expression, which results in the release of multiple pro-inflammatory mediators, including IL-4, IL-13, and TNF-α (55). The chemotherapeutic drug cisplatin (CDDP) provokes elevated levels of CKLF1 and NF-κB in HK-2 renal tubular epithelial cells and mouse kidneys, inducing inflammatory injury and nephrotoxicity. In contrast, Kanglaite (KLT) and hydroxytyrosol (HT) could partially reverse the adverse effects of CDDP by acting on the IκB-NF-κB complex and inhibiting the CKLF1-mediated NF-κB pathway (109). There is still a lack of research on the effect of CKLF1 in the NF-κB alternative pathway. However, this does not mean that CKLF1 acts only through the classical pathway. Some non-TNFR receptors have been shown to serve as stimulatory signals in the NF-κB alternative pathway (108). With the exploration of CKLF1 receptors, further studies may bring more insights into its pathogenic mechanisms. (**Figure 1**)

### 4.2 MAPK signaling pathway

MAPK pathways are highly conserved in all eukaryotic cells that consist of typical three-tier core signaling modules: MAP3Ks, MAP2Ks, and MAPKs. Through hierarchical phosphorylation across these modules, MAPK pathways can mediate gene transcription and regulate biological processes, including cell proliferation, differentiation, and apoptosis. Mammalian cells share three well-defined MAPK signaling pathways: ERK pathway, JNK pathway, and p38 pathway. Rather than being independent of each other, the three may share some upstream regulators and downstream target genes. MAPK pathways can be activated by many stimuli, including cytokines, growth factors, and pathogen-associated molecular patterns (PAMPs) (110).

**Figure 2:**
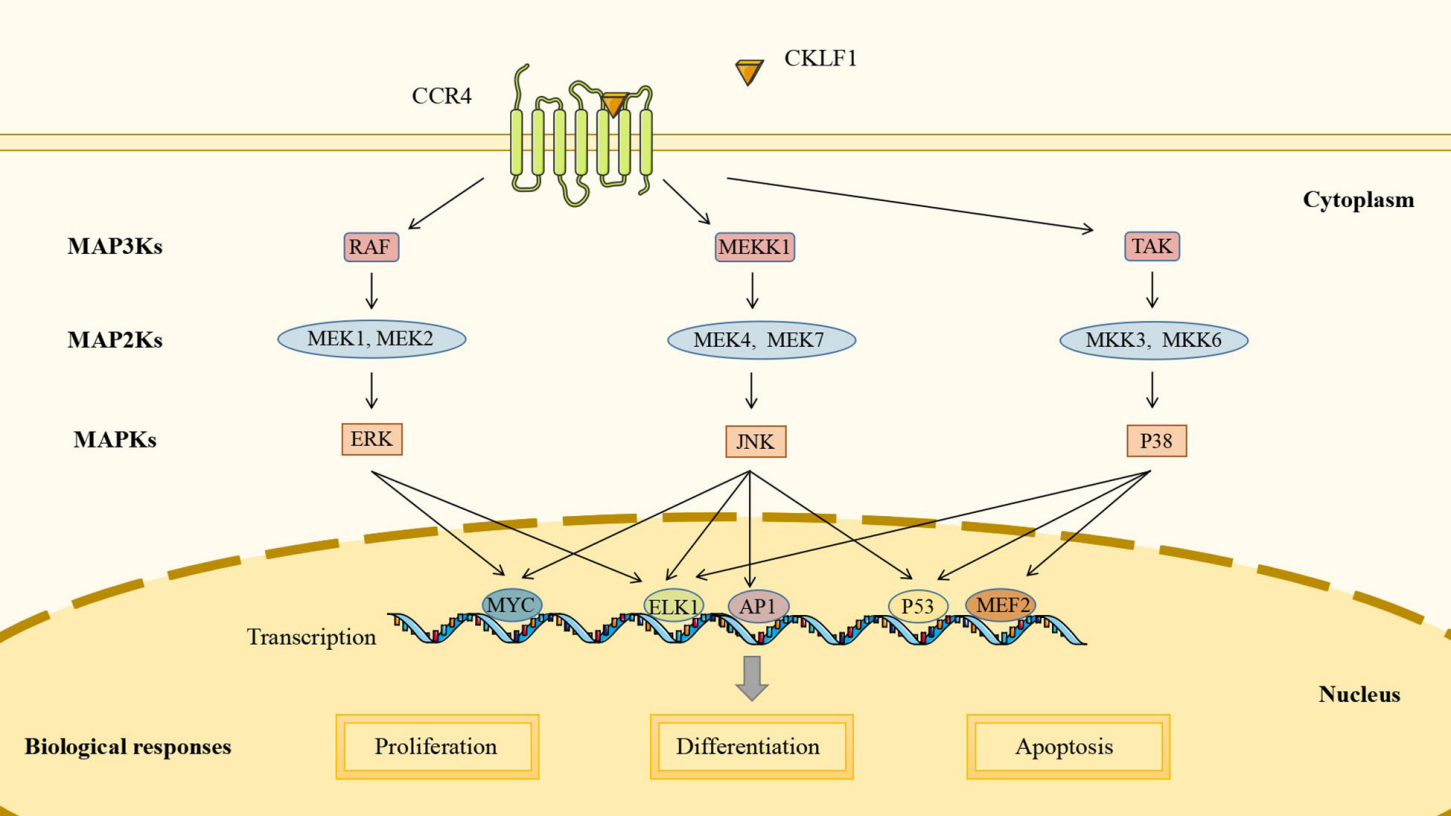
CKLF1 mediates cell proliferation, differentiation, and apoptosis by binding to CCR4 and activating the MAPK signaling pathway to induce intranuclear gene transcription.

Recent studies indicate that CKLF1 acts as a stimulatory factor to regulate the MAPK pathway and that the chemotactic activity of CKLF1 is partially dependent on the MAPK pathway, particularly neutrophil infiltration. A study utilizing an I/R model found that the expression of CKLF1 was significantly upregulated. The use of CKLF1 antibody significantly reduced the phosphorylation levels of ERK, JNK, and p38, inhibited the production of cytokines (TNF-α, MIP-2, and IL-1β) and the expression of adhesion factors (ICAM-1 and VCAM-1), and reduced the recruitment of neutrophils to the ischemic area (111). Fang et al. further demonstrated that treatment with the C19 peptide reduced the phosphorylation level of MAPK, with more pronounced effects on JNK and p38 (112). *In vitro* experiments with human umbilical vein endothelial cells have shown that the C19 peptide antagonized ERK and p38 signaling pathways, suggesting that it might be beneficial in psoriasis by inhibiting inflammatory infiltration and microvascular proliferation (65). In addition, it was shown that phosphorylation of JNK and p38 was involved in microglia M1 polarization, and CKLF1 could bind to CCR4 receptors on microglia. However, whether CKLF1 activates MAPK pathways through CCR4 receptors to mediate microglia polarization remains to be further investigated (113) (**Figure 2**).

### 4.3 IL-6/JAK/STAT3 signaling pathway

The IL-6/JAK/STAT3 signaling pathway is involved in regulating cell growth and differentiation, making it potentially critical in tumorigenesis and metastasis. Activation of the IL-6/JAK/STAT3 pathway often indicates poor prognosis in cancer patients (114, 115). It was established that IL-6 was highly expressed in the inflammatory and tumor microenvironment and bound to IL-6R and gp130 to form a heterohexameric complex to initiate signaling pathways and induce activation of gp130-associated JAKs. Mammals harbor four JAKs: JAK1, JAK2, JAK3, and TYK2, all of which can be activated in this pathway, mediating the phosphorylation of tyrosine residues to form protein docking sites. The same study also illustrated that STAT3 acted as an important substrate for JAK and was phosphorylated at tyrosine 705 (Y705). The phosphorylated STAT3 is then dimerized and translocated to the nucleus to regulate gene transcription (116).

**Figure 3:**
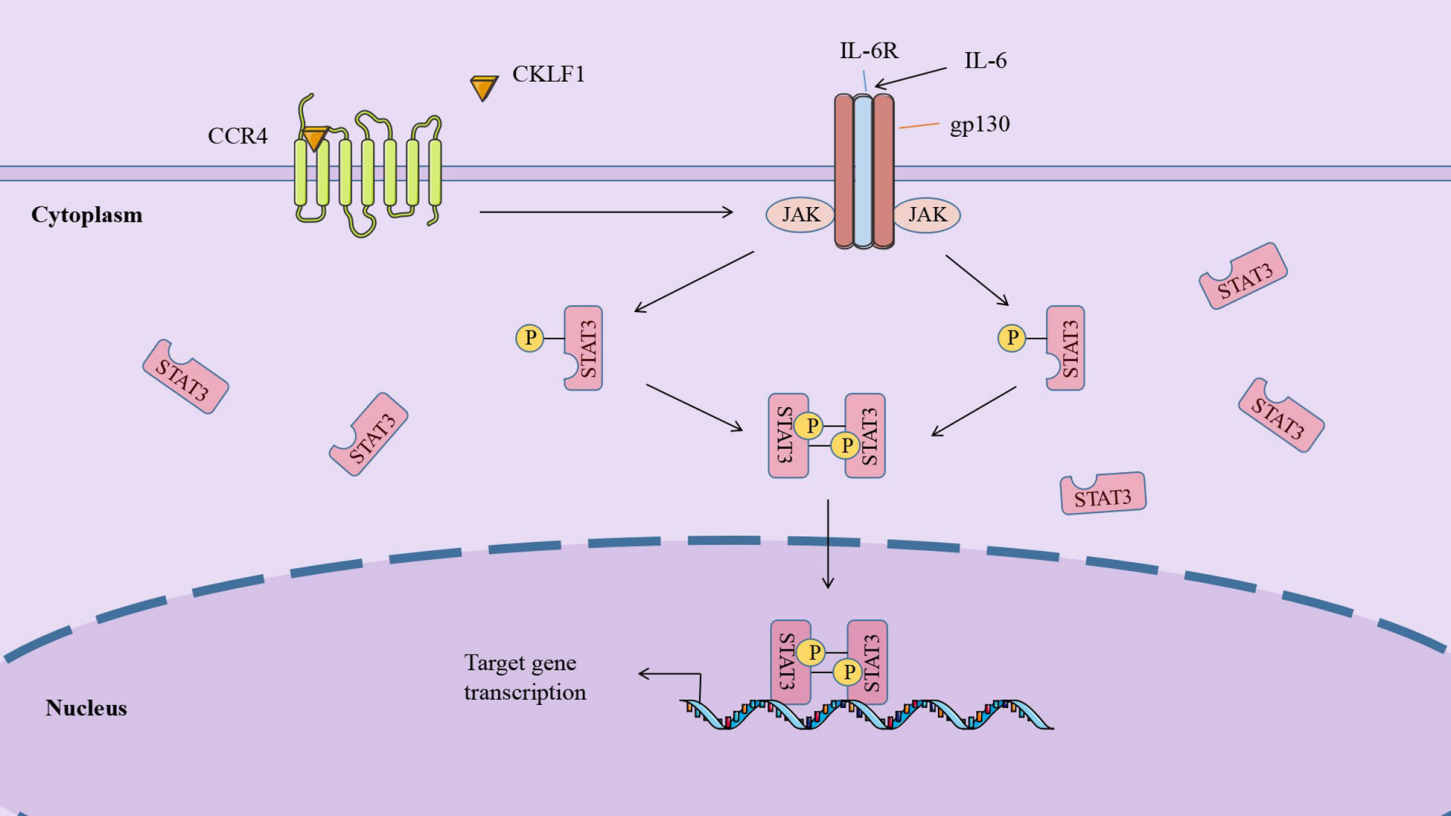
CKLF1 binds to CCR4 and induces the cell cycle regulatory gene expression through the IL-6/JAK/STAT3 signaling pathway, which is involved in tumor development.

The involvement of CKLF1 in tumor development may be related to its role in mediating the IL-6/JAK/STAT3 pathway. STAT3 is an important regulator in the metabolism of tumor cells. Overexpressed CKLF1 increases pyruvate kinase activity and promotes lactate production, which is accomplished by regulating STAT3. A study based on a hepatocellular carcinoma model demonstrated that CKLF1 regulates the tumor microenvironment by binding to CCR4 to initiate the IL-6/JAK/STAT3 signaling pathway, upregulating STAT3-related cytokines such as TNF-α and IL-17A, and inducing the expression of cell cycle regulatory genes including BCL-XL and cyclinsD1. In addition, these authors found that CKLF1 was able to affect the cell cycle of adriamycin (DOX)-transfected cells through the IL-6/JAK/STAT3 pathway, promoting proliferation in the G2/M phase and inhibiting DOX-induced apoptosis (74) (**Figure 3**).

### 4.4 PI3K/AKT signaling pathway

PI3K (an oncogene) is one component of the PI3K/AKT pathway, which is widely involved in tumors and immune-inflammatory diseases. The activation of tyrosine kinase receptors (RTKs), cytokine receptors, and G protein-coupled receptors (GPCRs) may act as upstream stimulatory signals of the PI3K/AKT pathway to activate PI3K (117). Activated PI3K catalyzes the conversion of PtdIns(4,5)P2 phosphorylation to PtdIns(3,4,5)P3, which acts as a second messenger to recruit AKT to specific sites on the cytoplasmic membrane. AKT is activated by the combined action of PDPK1 (phosphatidylinositol-dependent protein kinase 1) and mTORC2 (mammalian target of rapamycin complex 2) protein kinase to act on diverse downstream substrates and carry out biological functions like regulating cellular metabolism (118, 119).

**Figure 4:**
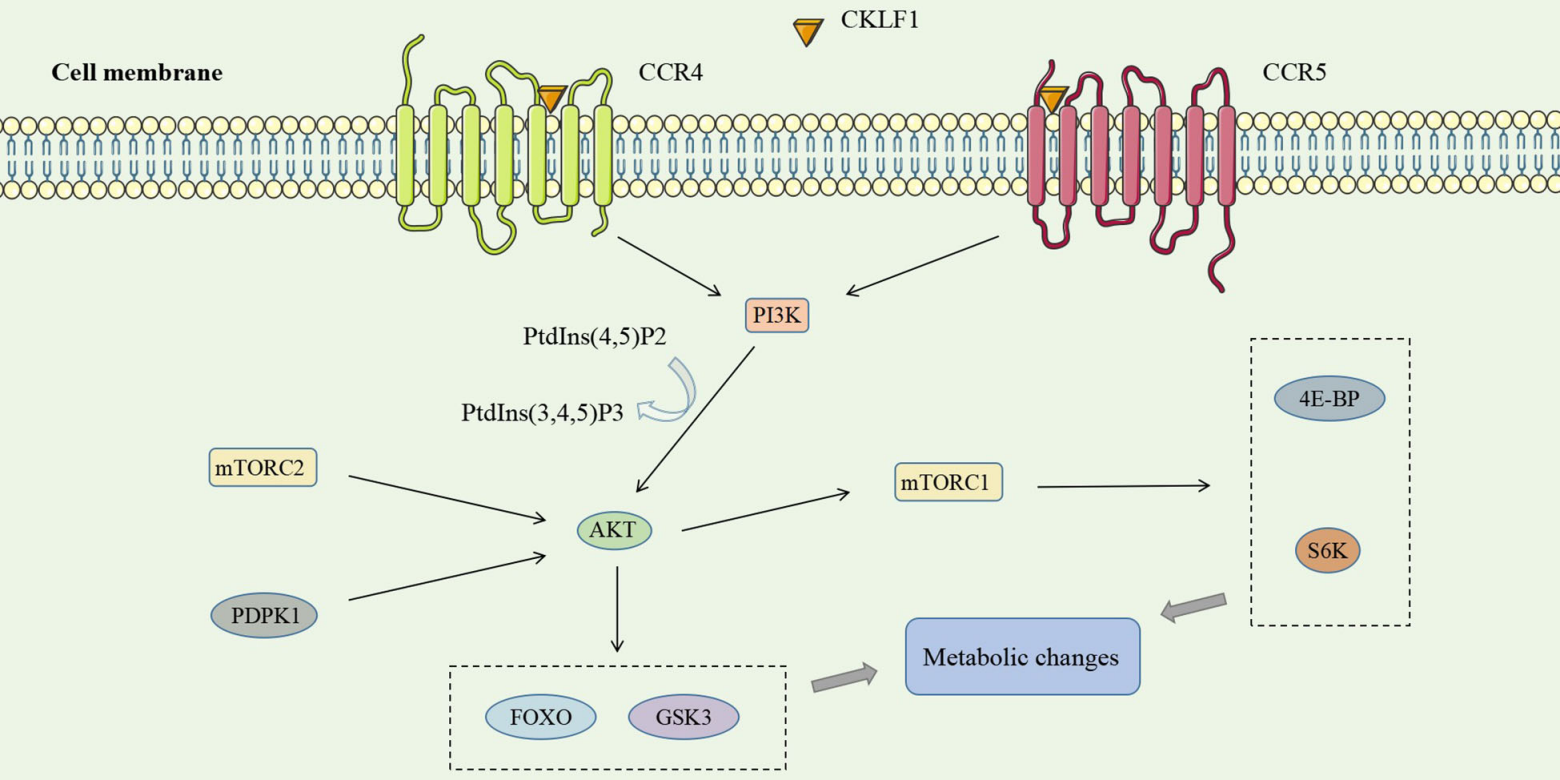
CKLF1 binds to both CCR4 and CCR5 and activates the PI3K/AKT signaling pathway, which plays a role in inflammatory injury through diverse downstream substrates of AKT and mTORC1.

CKLF1 has been found to contribute to inflammatory injury through a PI3K/AKT-dependent mechanism. VSMCs at lesion sites after vascular injury secrete CKLF1, which binds to the G protein-coupled receptor CCR4 to activate the PI3K/AKT signaling pathway, which regulates target gene transcription decreases susceptibility to apoptosis of G2/M phase cells and accelerates VSMC accumulation (120). Studies have revealed that CCR5 expression was upregulated after nerve injury (121, 122). In parallel, the CKLF1/CCR5 axis can activate downstream GSK3 *via* the AKT pathway and mediate neutrophil migration (36) (**Figure 4**).

### 4.5 Other signaling pathways

**Figure 5:**
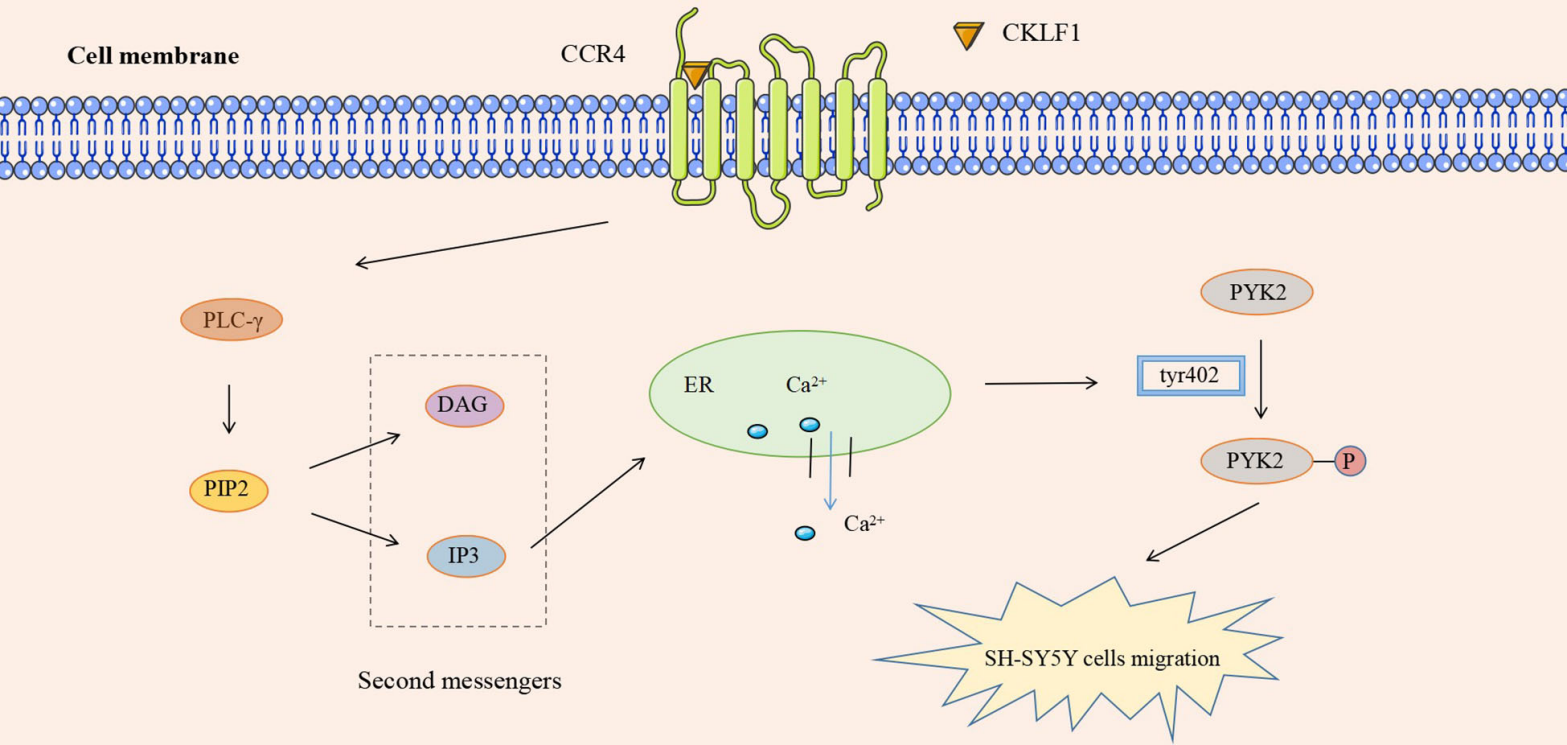
CKLF1 binds to CCR4 and induces the release of stored Ca2+ through the PYK2 pathway, activates autophosphorylation at the PYK2-tyr402 site, and regulates the migration of SH-SY5Y cells.

**Figure 6:**
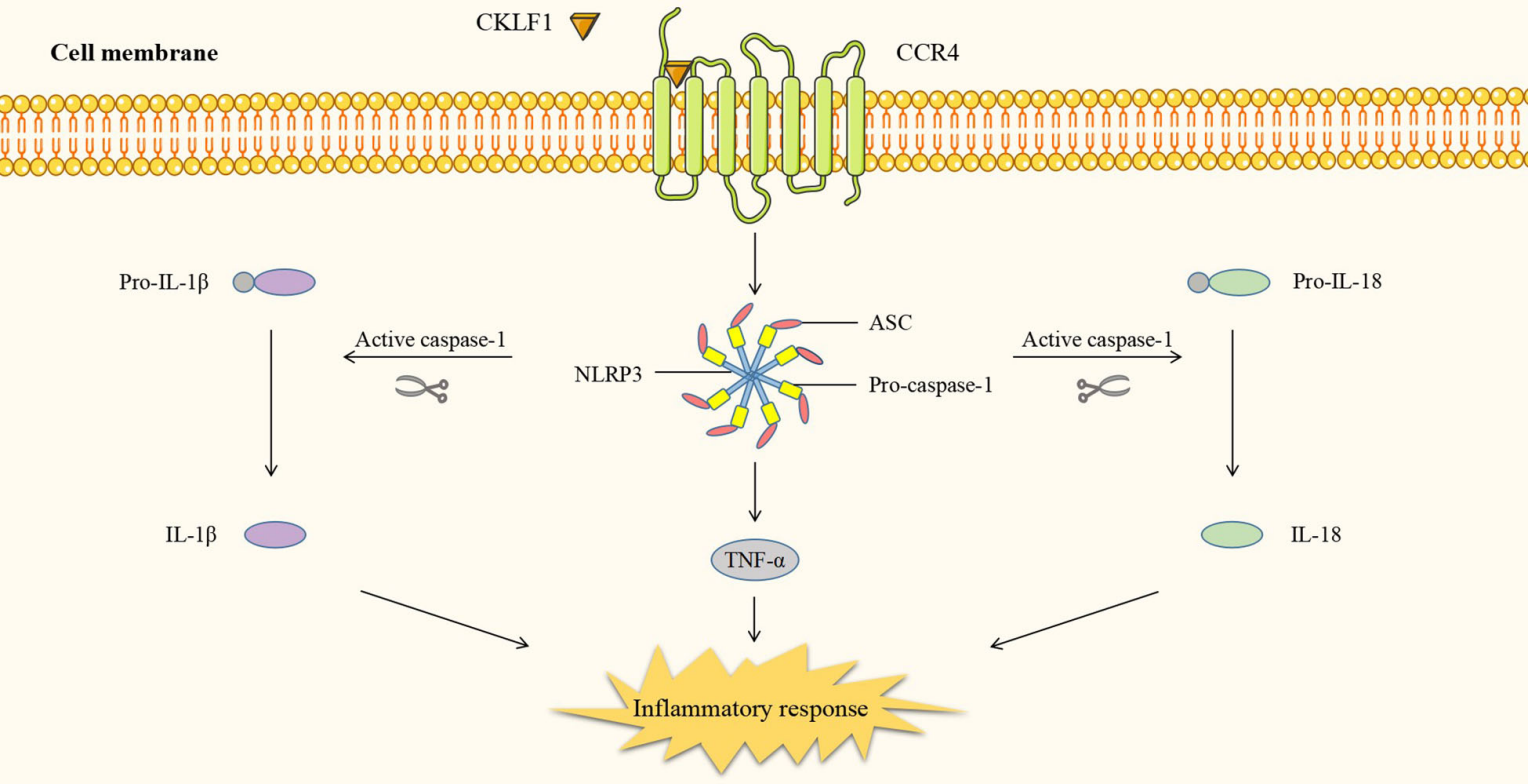
CKLF1 binds to CCR4 and mediates the activation of downstream NLRP3 inflammasomes. Activated caspase-1 induces maturation and release of pro-inflammatory cytokines and exacerbates inflammatory injury.

In addition to the four kinds of classical inflammatory signaling pathways, CKLF1 also functions by mediating other signaling pathways. Protein tyrosine kinase 2 (PYK2) is commonly present in actin filaments and is involved in the actin skeleton reorganization process. CKLF1 regulates cell migration-associated actin backbone reorganization *via* the non-extracellular Ca^2+^-dependent PYK2 pathway. A study found that CKLF1 in SH-SY5Y cells affected downstream phospholipase C-γ (PLC-γ) activity by binding to CCR4, triggering the hydrolysis of membrane phosphatidylinositides PIP2 and the production of second messengers DAG and IP3. IP3 then modulated the migration of SH-SY5Y cells by inducing the release of stored calcium ions and initiating autophosphorylation at the PYK2-tyr402 site (54) (**Figure 5**). NLRP3 inflammasomes are multimeric cytoplasmic protein complexes composed of the sensor protein NLRP3 linked to ASC and caspase-1, which are closely associated with oncological and metabolic diseases (123). CKLF1 was found to act on NLRP3-related signaling pathways and exert pro-inflammatory effects. CKLF1 expression was increased in the context of cerebral ischemic injury. Overexpressed CKLF1 bound to CCR4 and mediated the activation of downstream NLRP3 inflammasomes. The activation of caspase-1 then invoked the maturation and release of IL-1β, IL-18, and other pro-inflammatory cytokines, further exacerbating the inflammatory injury (124) (**Figure 6**).

### 4.6 Signaling crosstalk

**Figure 7:**
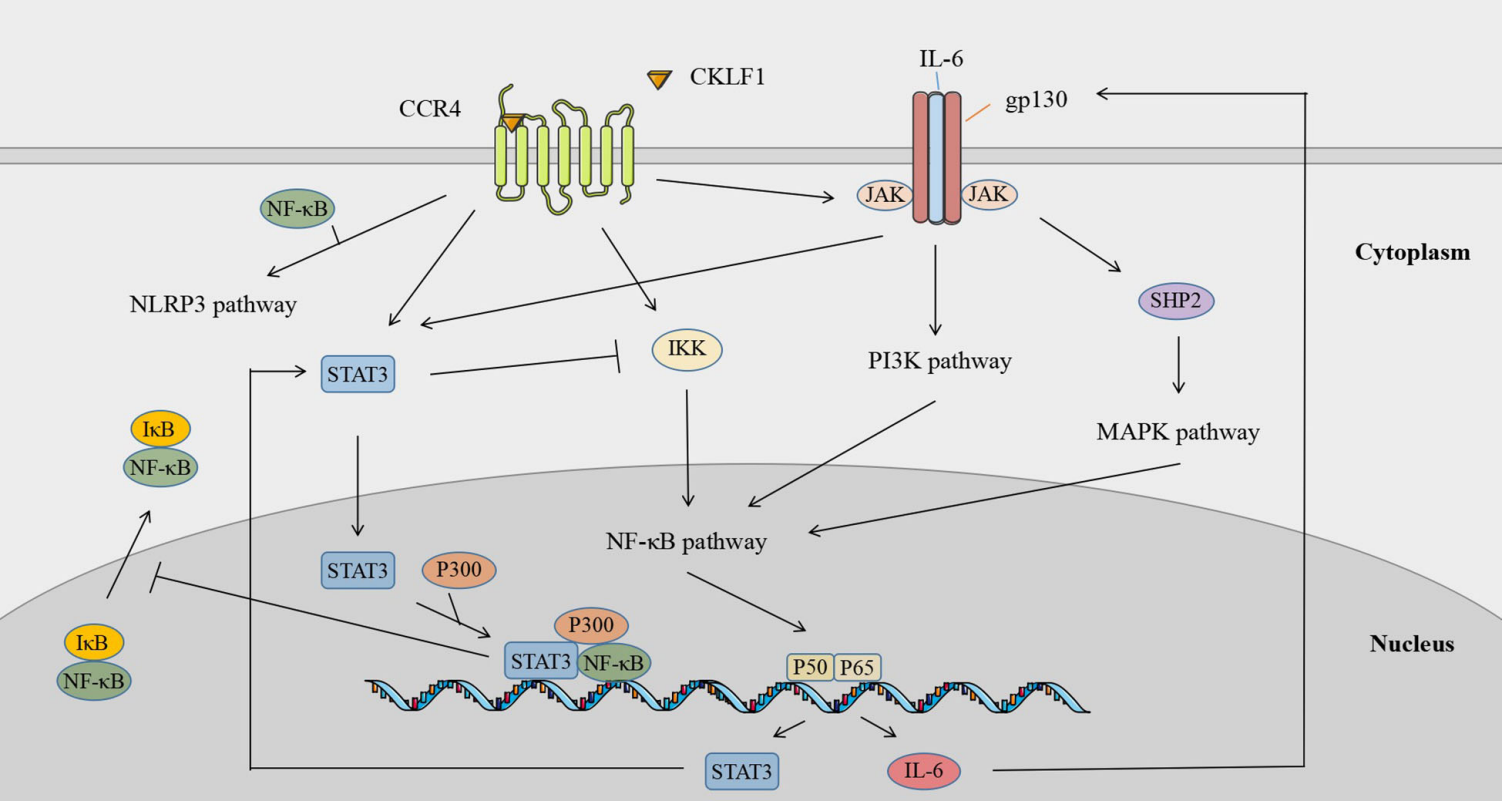
Signaling crosstalk across downstream pathways of CKLF1.

The different signaling pathways mediated by CKLF1 are not independent of each other but present signaling crosstalk that is highly dependent on the cellular environment. CKLF1 can simultaneously activate multiple signaling pathways involved in the pathogenic process. IL-6 and STAT3 are key components of the IL-6/JAK/STAT3 pathway and are also important products downstream of NF-κB. STAT3 acts on the CKLF1-mediated NF-κB pathway through two routes: a. STAT3 inhibits IKK activity and attenuates NF-κB pathway-associated Th1 cell immunity; b. activated STAT3 inhibits nuclear NF-κB-IκB complex migration to the cytoplasm through P300-mediated acetylation. Nuclear retention of NF-κB-IκB facilitates prolonged activation of the NF-κB pathway (125). In addition to being a stimulatory signal for the JAK/STAT3 pathway, IL-6 activates PI3K/AKT signaling and induces the MAPK cascade *via* SHP2 (tyrosine phosphatase containing the SH2 domain) (126). Meanwhile, CKLF1 links inflammatory stimuli and cellular responses by mediating MAPK (127–130) and PI3K/AKT (65) pathways to activate NF-κB transcription factors. In addition, NF-κB may be involved in the activation process of NLRP3 inflammasomes by CKLF1, of which the exact mechanism needs further clarifi (131) (**Figure 7**).

## 5 Treatments against CKLF1 mechanisms

Given the crucial role of CKLF1 in human diseases, treatments targeting CKLF1, CKLF1 receptors, and related pathways have become a hot research topic in the field of chemokines. (**Table 2**) Treatments targeting CKLF1 are highly specific and may be a preferred orientation for therapeutic options. A new 3-piperazinylcoumarin analogue (hereafter referred to as compound 41) has been identified as a potent antagonist of CKLF1. *In vitro* and mouse model experiments showed that compound 41 attenuated asthma pathological changes in CKLF1-transfected mice by inhibiting the binding of CKLF1 to CCR4 and affecting the activation of downstream NF-κB and other transduction pathways (56). By screening and optimization, investigators then obtained two derivatives of compound 41, IMMLG-5521 and IMM-H004. IMMLG-5521 inhibited CKLF1-induced eosinophil infiltration and TNF-α release and attenuated lung injury in rats (133). Subsequent studies showed that IMMLG-5521 inhibited CKLF1-induced eosinophil infiltration and TNF-α release, attenuating lung injury in rats. At the same time, IMM-H004 decreased the activation of microglia *via* the CKLF1/CCR4 axis, and played a protective role in central nervous system diseases such as Alzheimer’s disease and brain ischemia (48, 134, 135).

**Table 2:** Treatments against CKLF1 mechanisms.

Treatment strategy		Drugs	Limitations
Targeting CKLF1		Compound 41, IMMLG-5521,IMM-H004	Lack of clinical trial
Targeting CKLF1 receptors	CCR4	Compound 6b, compound 8a, 10E4, KW-0761	Species crossreactivity and pharmacokinetic properties remain to be solved
	CCR3	SB-328437 (132)	
	CCR5	Maraviroc (27)	
Targeting related pathways	NF-κB signaling pathway	WAY-169916, Bay-7082	Similar signaling processes in other cells are affected
	MAPK signaling pathway	SP600125, PD98059,SB203580	
	JAK/STAT3 signaling pathway	SOCS, CP-609550 , Stattic	
	PI3K/AKT signaling pathway	LY294002, wortmannin	

CCR4 is highly expressed in CKLF1-related diseases, and therapies targeting this receptor are being diligently developed. A study applied two identified CCR4 antagonists (hereafter referred to as compound 6b and compound 8a) to allergic rhinitis and asthmatic mice and observed that they somewhat alleviated symptoms (32). A CCR4-specific monoclonal antibody (referred to as 10E4 in this study) exhibits efficacy upon binding to the N-terminal end of CCR4 (136). KW-0761, a humanized monoclonal antibody targeting CCR4, has passed the phase I clinical trial and is intended to treat inflammatory diseases and tumors (132). In addition, the researches of antibodies targeting CCR3 and CCR5 have made some progress. Studies on applying the above antibodies in CKLF1-mediated disease are still lacking, but it could be hypothesized that they exert therapeutic effects by inhibiting the functional receptors of CKLF1. Classical inflammatory pathways dominate CKLF1 downstream signaling, and related inhibitors have been better characterized. For example, NF-κB transcription inhibitors WAY-169916 and Bay-7082, MAPK inhibitors SP600125, PD98059 and SB203580, JAK-STAT3 inhibitors SOCS, CP-609550, and Stattic, as well as PI3K pathway inhibitors LY294002 and Wortmannin (126, 137) can inhibit the biological function of CKLF1 by blocking signal transduction. Treatments targeting CKLF1 signaling pathways suffer from the drawback that similar signaling processes in other cells are affected. However, this drug category still provides a therapeutic option worthy of investigation.

## 6 Discussion

The discovery of CKLF1 opens up new insights into the immune-inflammatory mechanisms of diseases - excessive production of CKLF1 disrupts the balance of the immune environment to exert harmful effects. Current studies have been directed at the downstream signaling pathways of CKLF1, whereas clarity on its upstream regulatory mechanisms is still lacking. Here, we explored the possible upstream regulatory mechanisms of CKLF1 based on the existing studies on the regulatory mechanisms of chemokine family members. Gene-level regulation is mainly reflected in single nucleotide polymorphisms (SNPs). The CCL2-2518-A/G polymorphism has been extensively studied and proven to be a risk factor for Alzheimer’s disease (138, 139). SNPs of CXCL9-11 are closely associated with liver fibrosis (140). Apart from that, enhancers and promoters can activate transcription factors like NF-κB and AP-1 in response to inflammatory factors such as TNF-α and IL-6 and engage in the epigenetic regulation of chemokines (141). Post-transcriptional modifications, including DNA methylation and LncRNAs (long-stranded non-coding RNAs), were found to be associated with enhancer and promoter regions (142–144), which can exert a regulatory effect on chemokines by modulating inflammatory factor-responsive cis-acting elements. CHIP assay and luciferase assay showed that NF-κB could bind to the promoter region of the *CKLF1* (the core site of the *CKLF1* promoter is located at -238 to -249 bp) during ischemia and upregulate its expression (145). This finding confirms the epigenetic regulation mechanism of CKLF1. In recent years, cis-acting elements have emerged as favorable candidates for pharmacological interventions due to their broad activity and high level of target specificity against numerous pro-inflammatory genes. It is thus hypothesized that modulation of CKLF1 level by targeting the core site of the promoter of the CKLF1 gene may be beneficial for clinical treatments.

The main biological effects of CKLF1 are chemotactic activity and proliferation promotion. The good model of cerebral ischemia suggests that CKLF1 plays different roles in different stages of diseases, which may be a combination of the two biological effects. In the early stage of cerebral ischemia, CKLF1 recruits immune cells and aggravates brain injury; in the late stage of cerebral ischemia, CKLF1 promotes neurological recovery by promoting neuron and vascular regeneration (36). Current studies have focused on early stages of diseases, finding the time point at which the effect of CKLF1 changes is challenging. In addition, the role of CKLF1 on microglia polarization may be another reason for the difference. Microglia polarization has been found to be involved in the pathogenesis of a growing number of diseases. In immune-inflammatory diseases such as multiple sclerosis (146), M1 polarization in the early stage can induce the release of pro-inflammatory factors and exacerbate inflammatory response, while M2 polarization in the late stage is beneficial to inflammation regression and tissue repair. It has been confirmed that CKLF1 promotes M1 polarization in early cerebral ischemia, and future studies may further confirm the effect of CKLF1 on M2 polarization in the late stage of diseases. The diverse effects of CKLF1 in different stages of diseases suggest that the timing of using CKLF1 antagonists and agonists should be carefully selected.

## Author contributions

JF conceived and revised this review. YL took charge of the original manuscript writing. HY drew the figure and table. All authors contributed to the article and approved the submitted version.

## References

[1] Deuel TF Keim PS Farmer M Heinrikson RL. Amino acid sequence of human platelet factor 4. Proc Natl Acad Sci U.S.A. (1977) 74(6):2256–8. doi: 10.1073/pnas.74.6.2256 PMC432148 267922

[2] Anisowicz A Bardwell L Sager R. Constitutive overexpression of a growth-regulated gene in transformed Chinese hamster and human cells. Proc Natl Acad Sci U.S.A. (1987) 84(20):7188–92. doi: 10.1073/pnas.84.20.7188 PMC299255 2890161

[3] Sugano S Stoeckle MY Hanafusa H. Transformation by rous sarcoma virus induces a novel gene with homology to a mitogenic platelet protein. Cell (1987) 49(3):321–8. doi: 10.1016/0092-8674(87)90284-4 3032449

[4] Luster AD. Chemokines–chemotactic cytokines that mediate inflammation. N Engl J Med (1998) 338(7):436–45. doi: 10.1056/NEJM199802123380706 9459648

[5] Charo IF Ransohoff RM. The many roles of chemokines and chemokine receptors in inflammation. N Engl J Med (2006) 354(6):610–21. doi: 10.1056/NEJMra052723 16467548

[6] Cao S Liu M Sehrawat TS Shah VH. Regulation and functional roles of chemokines in liver diseases. Nat Rev Gastroenterol Hepatol (2021) 18(9):630–47. doi: 10.1038/s41575-021-00444-2 PMC9036964 33976393

[7] Zlotnik A Yoshie O. Chemokines: a new classification system and their role in immunity. Immunity (2000) 12(2):121–7. doi: 10.1016/S1074-7613(00)80165-X 10714678

[8] Han W Lou Y Tang J Zhang Y Chen Y Li Y. Molecular cloning and characterization of chemokine-like factor 1 (CKLF1), a novel human cytokine with unique structure and potential chemotactic activity. Biochem J (2001) 357(Pt 1):127–35. doi: 10.1042/bj3570127 PMC1221935 11415443

[9] Han W Ding P Xu M Wang L Rui M Shi S. Identification of eight genes encoding chemokine-like factor superfamily members 1-8 (CKLFSF1-8) by in silico cloning and experimental validation. Genomics (2003) 81(6):609–17. doi: 10.1016/S0888-7543(03)00095-8 12782130

[10] Zhong J Wang Y Qiu X Mo X Liu Y Li T. Characterization and expression profile of CMTM3/CKLFSF3. J Biochem Mol Biol (2006) 39:537–45. doi: 10.5483/BMBRep.2006.39.5.537 17002874

[11] Cao L Yang C Zhu B Zhang G Zhao N Liu X. A novel protein CMTM1-v5 specifically induced human lymphoma cells apoptosis *in vitro* and *in vivo* . Exp Cell Res (2019) 385:111623. doi: 10.1016/j.yexcr.2019.111623 31542285

[12] Kumar S Kang H Park E Park HS Lee K. The expression of CKLFSF2B is regulated by GATA1 and CREB in the leydig cells, which modulates testicular steroidogenesis. Biochim Biophys Acta Gene Regul Mech (2018) 1861:1063–75. doi: 10.1016/j.bbagrm.2018.10.002 30321752

[13] Chrifi I Louzao-Martinez L Brandt M van Dijk CGM Burgisser P Zhu C. CMTM3 (CKLF-like marvel transmembrane domain 3) mediates angiogenesis by regulating cell surface availability of VE-cadherin in endothelial adherens junctions. Arterioscler Thromb Vasc Biol (2017) 37:1098–114. doi: 10.1161/ATVBAHA.116.308792 28428220

[14] Chui NN Cheu JW Yuen VW Chiu DK Goh CC Lee D. Inhibition of CMTM4 sensitizes cholangiocarcinoma and hepatocellular carcinoma to T cell-mediated antitumor immunity through PD-L1. Hepatol Commun (2022) 6:178–93. doi: 10.1002/hep4.1682 PMC8710793 34558800

[15] Li H Liu Y Chen L Zhou JJ Chen DR Li SJ. CMTM4 regulates epithelial-mesenchymal transition and PD-L1 expression in head and neck squamous cell carcinoma. Mol Carcinog (2021) 60:556–66. doi: 10.1002/mc.23323 34061408

[16] Mezzadra R Sun C Jae L Gomez-Eerland R de Vries E Wu W. Identification of CMTM6 and CMTM4 as PD-L1 protein regulators. Nature (2017) 549:106–10. doi: 10.1038/nature23669 PMC6333292 28813410

[17] Zhang J Liu T Chen X Liang WY Feng XR Wang L. Validation of aspirin response-related transcripts in patients with coronary artery disease and preliminary investigation on CMTM5 function. Gene (2017) 624:56–65. doi: 10.1016/j.gene.2017.04.041 28457985

[18] Buscham TJ Eichel-Vogel MA Steyer AM Jahn O Strenzke N Dardawal R. Progressive axonopathy when oligodendrocytes lack the myelin protein CMTM5. Elife (2022) 11. undefined. doi: 10.7554/eLife.75523 PMC8916772 35274615

[19] Wu J. CMTM5/7 are biomarkers and prognostic factors in human breast carcinoma. Cancer biomark (2020) 29:89–99. doi: 10.3233/CBM-191226 32568178 PMC12662502

[20] Wang H Fan Y Chen W Lv Z Wu S Xuan Y. Loss of CMTM6 promotes DNA damage-induced cellular senescence and antitumor immunity. Oncoimmunology (2022) 11:2011673. doi: 10.1080/2162402X.2021.2011673 35024247 PMC8747516

[21] Gao D Hu H Fang Z Huo F Wang HR Xu KX. Research advances in chemokine-like factor super family member 8. Zhongguo Yi Xue Ke Xue Yuan Xue Bao (2016) 38:746–9. doi: 10.3881/j.issn,1000-503X.2016.06.021 28065246

[22] Zhang S Pei X Hu H Zhang W Mo X Song Q. Functional characterization of the tumor suppressor CMTM8 and its association with prognosis in bladder cancer. Tumour Biol (2016) 37:6217–25. doi: 10.1007/s13277-015-4508-6 26615421

[23] Lou Y Xia D Han W Wang Y Li X Li Y. Molecular cloning and characterization of rat chemokine-like factor 1 and 2. Gene (2003) 307:125–32. doi: 10.1016/S0378-1119(03)00450-5 12706894

[24] Rui M Xia D Zhang Y Han W Wang L Ding P. Molecular cloning and characterization of four isoforms of mCKLF, mouse homologues of human chemokine-like factor. Mol Biol Rep (2003) 30(4):229–37. doi: 10.1023/A:1026308129769 14672409

[25] Yoshie O Matsushima K. CCR4 and its ligands: from bench to bedside. Int Immunol (2015) 27(1):11–20. doi: 10.1093/intimm/dxu079 25087232

[26] Wang Y Zhang Y Yang X Han W Liu Y Xu Q. Chemokine-like factor 1 is a functional ligand for CC chemokine receptor 4 (CCR4). Life Sci (2006) 78(6):614–21. doi: 10.1016/j.lfs.2005.05.070 PMC7126766 16137713

[27] Hutchings CJ Koglin M Olson WC Marshall FH. Opportunities for therapeutic antibodies directed at G-protein-coupled receptors. Nat Rev Drug Discovery (2017) 16(9):661. doi: 10.1038/nrd.2017.91 28860586

[28] Andrews G Jones C Wreggett KA. An intracellular allosteric site for a specific class of antagonists of the CC chemokine G protein-coupled receptors CCR4 and CCR5. Mol Pharmacol (2008) 73(3):855–67. doi: 10.1124/mol.107.039321 18042736

[29] Wang Y Zhang Y Han W Li D Tian L Yin C. Two c-terminal peptides of human CKLF1 interact with the chemokine receptor CCR4. Int J Biochem Cell Biol (2008) 40(5):909–19. doi: 10.1016/j.biocel.2007.10.028 18069042

[30] Schwarz MK Wells TN. New therapeutics that modulate chemokine networks. Nat Rev Drug Discovery (2002) 1(5):347–58. doi: 10.1038/nrd795 12120410

[31] Fulkerson PC Fischetti CA McBride ML Hassman LM Hogan SP Rothenberg ME. A central regulatory role for eosinophils and the eotaxin/CCR3 axis in chronic experimental allergic airway inflammation. Proc Natl Acad Sci U.S.A. (2006) 103(44):16418–23. doi: 10.1073/pnas.0607863103 PMC1637597 17060636

[32] Zheng Y Guo C Zhang Y Qi H Sun Q Xu E. Alleviation of murine allergic rhinitis by C19, a c-terminal peptide of chemokine-like factor 1 (CKLF1). Int Immunopharmacol (2011) 11(12):2188–93. doi: 10.1016/j.intimp.2011.09.017 22001899

[33] Bachelerie F Ben-Baruch A Burkhardt AM Combadiere C Farber JM Graham GJ. International union of basic and clinical pharmacology. [corrected]. LXXXIX. update on the extended family of chemokine receptors and introducing a new nomenclature for atypical chemokine receptors. Pharmacol Rev (2014) 66(1):1–79. doi: 10.1124/pr.113.007724 24218476 PMC3880466

[34] Smyth DJ Plagnol V Walker NM Cooper JD Downes K Yang JH. Shared and distinct genetic variants in type 1 diabetes and celiac disease. N Engl J Med (2008) 359(26):2767–77. doi: 10.1056/NEJMoa0807917 PMC2840835 19073967

[35] Lee B Sharron M Montaner LJ Weissman D Doms RW. Quantification of CD4, CCR5, and CXCR4 levels on lymphocyte subsets, dendritic cells, and differentially conditioned monocyte-derived macrophages. Proc Natl Acad Sci U.S.A. (1999) 96(9):5215–20. doi: 10.1073/pnas.96.9.5215 PMC21844 10220446

[36] Chen C Chu SF Ai QD Zhang Z Chen NH. CKLF1/CCR5 axis is involved in neutrophils migration of rats with transient cerebral ischemia. Int Immunopharmacol (2020) 85:106577. doi: 10.1016/j.intimp.2020.106577 32446198

[37] Banchereau J Steinman RM. Dendritic cells and the control of immunity. Nature (1998) 392(6673):245–52. doi: 10.1038/32588 9521319

[38] Yamauchi A Dai SY Nakagawa R Kashio Y Abe H Katoh S. [Galectin-9 induces maturation of human monocyte-derived dendritic cells]. Nihon Rinsho Meneki Gakkai Kaishi (2005) 28(6):381–8. doi: 10.2177/jsci.28.381 16394641

[39] Nakahara T Moroi Y Uchi H Furue M. Differential role of MAPK signaling in human dendritic cell maturation and Th1/Th2 engagement. J Dermatol Sci (2006) 42(1):1–11. doi: 10.1016/j.jdermsci.2005.11.004 16352421

[40] Shao L Li T Mo X Majdic O Zhang Y Seyerl M. Expressional and functional studies of CKLF1 during dendritic cell maturation. Cell Immunol (2010) 263(2):188–95. doi: 10.1016/j.cellimm.2010.03.015 20392439

[41] Li T Zhong J Chen Y Qiu X Zhang T Ma D. Expression of chemokine-like factor 1 is upregulated during T lymphocyte activation. Life Sci (2006) 79(6):519–24. doi: 10.1016/j.lfs.2006.01.042 16522323

[42] Kim CH Lim HW Kim JR Rott L Hillsamer P Butcher EC. Unique gene expression program of human germinal center T helper cells. Blood (2004) 104(7):1952–60. doi: 10.1182/blood-2004-03-1206 15213097

[43] Feng XR Hong T Gong YJ Bu DF Yuan JY Xue L. [*In vivo* transfer of human chemokine-like factor 1 gene increases peripheral blood CD34+ stem cells after myocardial infarction in rats]. Beijing Da Xue Xue Bao Yi Xue Ban (2006) 38(6):592–6. doi: 10.1016/S1567-5688(06)80364-8 17173078

[44] Zhang T Zhang X Yu W Chen J Li Q Jiao Y. Effects of chemokine-like factor 1 on vascular smooth muscle cell migration and proliferation in vascular inflammation. Atherosclerosis (2013) 226(1):49–57. doi: 10.1016/j.atherosclerosis.2012.09.023 23102782

[45] Cai X Deng J Ming Q Cai H Chen Z. Chemokine-like factor 1: A promising therapeutic target in human diseases. Exp Biol Med (Maywood) (2020) 245(16):1518–28. doi: 10.1177/1535370220945225 PMC7553088 32715782

[46] Nam KW Kim TJ Lee JS Kwon HM Lee YS Ko SB. High neutrophil-to-Lymphocyte ratio predicts stroke-associated pneumonia. Stroke (2018) 49(8):1886–92. doi: 10.1161/STROKEAHA.118.021228 29967014

[47] Kong LL Wang ZY Han N Zhuang XM Wang ZZ Li H. Neutralization of chemokine-like factor 1, a novel c-c chemokine, protects against focal cerebral ischemia by inhibiting neutrophil infiltration *via* MAPK pathways in rats. J Neuroinflamm (2014) 11:112. doi: 10.1186/1742-2094-11-112 PMC4080607 24946684

[48] Chen C Ai Q Chu S Zhang Z Zhou X Luo P. IMM-H004 protects against oxygen-glucose deprivation/reperfusion injury to BV2 microglia partly by modulating CKLF1 involved in microglia polarization. Int Immunopharmacol (2019) 70:69–79. doi: 10.1016/j.intimp.2019.02.012 30785093

[49] Chen C Chu SF Ai QD Zhang Z Guan FF Wang SS. CKLF1 aggravates focal cerebral ischemia injury at early stage partly by modulating Microglia/Macrophage toward M1 polarization through CCR4. Cell Mol Neurobiol (2019) 39(5):651–69. doi: 10.1007/s10571-019-00669-5 PMC11462892 30982091

[50] Zhou Y Li HQ Lu L Fu DL Liu AJ Li J H. Ginsenoside Rg1 provides neuroprotection against blood brain barrier disruption and neurological injury in a rat model of cerebral ischemia/reperfusion through downregulation of aquaporin 4 expression. Phytomedicine (2014) 21(7):998–1003. doi: 10.1016/j.phymed.2013.12.005 24462216

[51] Kong LL Wang ZY Hu JF Yuan YH Li H Chen NH. Inhibition of chemokine-like factor 1 improves blood-brain barrier dysfunction in rats following focal cerebral ischemia. Neurosci Lett (2016) 627:192–8. doi: 10.1016/j.neulet.2016.06.003 27283776

[52] Wang ZZ Zhang Y Yuan YH Chen NH. Developmental expression of chemokine-like factor 1, a novel member of chemokines family, in postnatal rat cerebral cortex. Neurosci Lett (2012) 519(1):51–5. doi: 10.1016/j.neulet.2012.05.019 22587964

[53] Wang ZZ Yuan YH Zhang Y Wang XF Chu SF Han N. Chemokine-like factor 1 promotes the migration of rat primary cortical neurons by the induction of actin polymerization. Neuroreport (2014) 25(15):1221–6. doi: 10.1097/WNR.0000000000000252 25144394

[54] Wang ZZ Li G Chen XY Zhao M Yuan YH Wang XL. Chemokine-like factor 1, a novel cytokine, induces nerve cell migration through the non-extracellular Ca2+-dependent tyrosine kinases pathway. Brain Res (2010) 308:24–34. doi: 10.1016/j.brainres.2009.10.047 19857473

[55] Li G Li GY Wang ZZ Ji HJ Wang DM Hu JF. The chemokine-like factor 1 induces asthmatic pathological change by activating nuclear factor-κB signaling pathway. Int Immunopharmacol (2014) 20(1):81–8. doi: 10.1016/j.intimp.2014.02.014 24583145

[56] Li G Wang D Sun M Li G Hu J Zhang Y. Discovery and optimization of novel 3-piperazinylcoumarin antagonist of chemokine-like factor 1 with oral antiasthma activity in mice. J Med Chem (2010) 53(4):1741–54. doi: 10.1021/jm901652p 20099827

[57] Tian L Li W Wang J Zhang Y Zheng Y Qi H. The CKLF1-C19 peptide attenuates allergic lung inflammation by inhibiting CCR3- and CCR4-mediated chemotaxis in a mouse model of asthma. Allergy (2011) 66(2):287–97. doi: 10.1111/j.1398-9995.2010.02478.x 21208220

[58] Zhang Y Wu Y Qi H Xiao J Gong H Zhang Y. A new antagonist for CCR4 attenuates allergic lung inflammation in a mouse model of asthma. Sci Rep (2017) 7(1):15038. doi: 10.1038/s41598-017-11868-9 29118379 PMC5678437

[59] Tan YX Han WL Chen YY Ouyang NT Tang Y Li F. Chemokine-like factor 1, a novel cytokine, contributes to airway damage, remodeling and pulmonary fibrosis. Chin Med J (Engl) (2004) 117(8):1123–9. 15361282

[60] Tao K Tang X Wang B Li RJ Zhang BQ Lin JH. Distinct expression of chemokine-like factor 1 in synovium of osteoarthritis, rheumatoid arthritis and ankylosing spondylitis. J Huazhong Univ Sci Technolog Med Sci (2016) 36(1):70–6. doi: 10.1007/s11596-016-1544-4 26838743

[61] Yildirim K Karatay S Melikoglu MA Gureser G Ugur M Senel K. Associations between acute phase reactant levels and disease activity score (DAS28) in patients with rheumatoid arthritis. Ann Clin Lab Sci (2004) 34(4):423–6. 15648784

[62] Pearle AD Scanzello CR George S Mandl LA DiCarlo EF Peterson M. Elevated high-sensitivity c-reactive protein levels are associated with local inflammatory findings in patients with osteoarthritis. Osteoarthritis Cartilage (2007) 15(5):516–23. doi: 10.1016/j.joca.2006.10.010 17157039

[63] de Andrade KR de Castro GR Vicente G da Rosa JS Nader M Pereira IA. Evaluation of circulating levels of inflammatory and bone formation markers in axial spondyloarthritis. Int Immunopharmacol (2014) 21(2):481–6. doi: 10.1016/j.intimp.2014.05.031 24925756

[64] Tan Y Wang Y Li L Xia J Peng S He Y. Chemokine-like factor 1-derived c-terminal peptides induce the proliferation of dermal microvascular endothelial cells in psoriasis. PloS One (2015) 10(4):e0125073. doi: 10.1371/journal.pone.0125073 25915746 PMC4410955

[65] Zheng Y Wang Y Zhang X Tan Y Peng S Chen L. C19, a c-terminal peptide of CKLF1, decreases inflammation and proliferation of dermal capillaries in psoriasis. Sci Rep (2017) 7(1):13890. doi: 10.1038/s41598-017-13799-x 29066845 PMC5655640

[66] Ji Y Zhang H Yuan H Yang GP Zhang K Xie LH. [Expression of chemokine like factor-1 in nephridial tissue of lupus nephritis]. Zhong Nan Da Xue Xue Bao Yi Xue Ban (2007) 32(3):490–3. 17611331

[67] Lood C Amisten S Gullstrand B Jönsen A Allhorn M Truedsson L. Platelet transcriptional profile and protein expression in patients with systemic lupus erythematosus: up-regulation of the type I interferon system is strongly associated with vascular disease. Blood (2010) 116(11):1951–7. doi: 10.1182/blood-2010-03-274605 20538795

[68] Chowdhury MH Nagai A Terashima M Sheikh A Murakawa Y Kobayashi S. Chemokine-like factor expression in the idiopathic inflammatory myopathies. Acta Neurol Scand (2008) 118(2):106–14. doi: 10.1111/j.1600-0404.2007.00990.x 18294340

[69] Zhang T Qiao Z Chen F Zhang X Xiong J Jia X. Antagonistic effect of C19 on migration of vascular smooth muscle cells and intimal hyperplasia induced by chemokine-like factor 1. Mol Biol Rep (2013) 40(4):2939–46. doi: 10.1007/s11033-012-2309-1 23203409

[70] Duan Y Zhang Y Qu C Yu W Tana Shen C. CKLF1 aggravates neointimal hyperplasia by inhibiting apoptosis of vascular smooth muscle cells through PI3K/AKT/NF-κB signaling. BioMed Pharmacother (2019) 117:108986. doi: 10.1016/j.biopha.2019.108986 31387172

[71] Liu X Qu C Zhang Y Fang J Teng L Zhang R. Chemokine-like factor 1 (CKLF1) aggravates neointimal hyperplasia through activating the NF-κB/VCAM-1 pathway. FEBS Open Bio (2020) 10(9):1880–90. doi: 10.1002/2211-5463.12942 PMC7459414 32741140

[72] Li J Bao X Li Y Wang Y Zhao Z Jin X. Study of the functional mechanisms of osteopontin and chemokine-like factor 1 in the development and progression of abdominal aortic aneurysms in rats. Exp Ther Med (2016) 12(6):4007–11. doi: 10.3892/etm.2016.3891 PMC5228271 28101179

[73] Zhang M Xu Y Liu Y Cheng Y Zhao P Liu H. Chemokine-like factor 1 (CKLF-1) is overexpressed in keloid patients: A potential indicating factor for keloid-predisposed individuals. Med (Baltimore) (2016) 95(11):e3082. doi: 10.1097/MD.0000000000003082 PMC4839923 26986142

[74] Liu Y Liu L Zhou Y Zhou P Yan Q Chen X. CKLF1 enhances inflammation-mediated carcinogenesis and prevents doxorubicin-induced apoptosis *via* IL6/STAT3 signaling in HCC. Clin Cancer Res (2019) 25(13):4141–54. doi: 10.1158/1078-0432.CCR-18-3510 30918019

[75] Gasbarrino K. Di iorio d and daskalopoulou SS. importance of sex and gender in ischaemic stroke and carotid atherosclerotic disease. Eur Heart J (2022) 43(6):460–73. doi: 10.1093/eurheartj/ehab756 PMC8830529 34849703

[76] Lakhan SE Kirchgessner A Hofer M. Inflammatory mechanisms in ischemic stroke: therapeutic approaches. J Transl Med (2009) 7:97. doi: 10.1186/1479-5876-7-97 19919699 PMC2780998

[77] David S Kroner A. Repertoire of microglial and macrophage responses after spinal cord injury. Nat Rev Neurosci (2011) 12(7):388–99. doi: 10.1038/nrn3053 21673720

[78] Parsa R Lund H Tosevski I Zhang XM Malipiero U Beckervordersandforth J. TGFβ regulates persistent neuroinflammation by controlling Th1 polarization and ROS production *via* monocyte-derived dendritic cells. Glia (2016) 64(11):1925–37. doi: 10.1002/glia.23033 PMC5053226 27479807

[79] Guarnieri FC de Chevigny A Falace A Cardoso C. Disorders of neurogenesis and cortical development. Dialogues Clin Neurosci (2018) 20(4):255–66. doi: 10.31887/DCNS.2018.20.4/ccardoso PMC6436956 30936766

[80] Merino JJ Bellver-Landete V Oset-Gasque MJ Cubelos B. CXCR4/CXCR7 molecular involvement in neuronal and neural progenitor migration: focus in CNS repair. J Cell Physiol (2015) 230(1):27–42. doi: 10.1002/jcp.24695 24913264

[81] Lagane B Chow KY Balabanian K Levoye A Harriague J Planchenault T. CXCR4 dimerization and beta-arrestin-mediated signaling account for the enhanced chemotaxis to CXCL12 in WHIM syndrome. Blood (2008) 112(1):34–44. doi: 10.1182/blood-2007-07-102103 18436740

[82] Tiveron MC Cremer H. CXCL12/CXCR4 signalling in neuronal cell migration. Curr Opin Neurobiol (2008) 18(3):237–44. doi: 10.1016/j.conb.2008.06.004 18644448

[83] Pritchett J Wright C Zeef L Nadarajah B. Stromal derived factor-1 exerts differential regulation on distinct cortical cell populations *in vitro* . BMC Dev Biol (2007) 7:31. doi: 10.1186/1471-213X-7-31 17425785 PMC1854892

[84] Imai T Baba M Nishimura M Kakizaki M Takagi S Yoshie O. The T cell-directed CC chemokine TARC is a highly specific biological ligand for CC chemokine receptor 4. J Biol Chem (1997) 272(23):15036–42. doi: 10.1074/jbc.272.23.15036 9169480

[85] Imai T Chantry D Raport CJ Wood CL Nishimura M Godiska R. Macrophage-derived chemokine is a functional ligand for the CC chemokine receptor 4. J Biol Chem (1998) 273(3):1764–8. doi: 10.1074/jbc.273.3.1764 9430724

[86] Imai T Nagira M Takagi S Kakizaki M Nishimura M Wang J. Selective recruitment of CCR4-bearing Th2 cells toward antigen-presenting cells by the CC chemokines thymus and activation-regulated chemokine and macrophage-derived chemokine. Int Immunol (1999) 11(1):81–8. doi: 10.1093/intimm/11.1.81 10050676

[87] Honjo A Ogawa H Azuma M Tezuka T Sone S Biragyn A. Targeted reduction of CCR4^+^ cells is sufficient to suppress allergic airway inflammation. Respir Investig (2013) 51(4):241–9. doi: 10.1016/j.resinv.2013.04.007 PMC5846619 24238232

[88] Bisset LR Schmid-Grendelmeier P. Chemokines and their receptors in the pathogenesis of allergic asthma: progress and perspective. Curr Opin Pulm Med (2005) 11(1):35–42. doi: 10.1097/01.mcp.0000144502.50149.e0 15591886

[89] Lee KS Lee HK Hayflick JS Lee YC Puri KD. Inhibition of phosphoinositide 3-kinase delta attenuates allergic airway inflammation and hyperresponsiveness in murine asthma model. FASEB J (2006) 20(3):455–65. doi: 10.1096/fj.05-5045com 16507763

[90] Stone KD Prussin C Metcalfe DD. IgE, mast cells, basophils, and eosinophils. J Allergy Clin Immunol (2010) 125(2 Suppl 2):S73–80. doi: 10.1016/j.jaci.2009.11.017 PMC2847274 20176269

[91] König A Krenn V Toksoy A Gerhard N Gillitzer R. Mig, GRO alpha and RANTES messenger RNA expression in lining layer, infiltrates and different leucocyte populations of synovial tissue from patients with rheumatoid arthritis, psoriatic arthritis and osteoarthritis. Virchows Arch (2000) 436(5):449–58. doi: 10.1007/s004280050472 10881738

[92] Nanki T Lipsky PE. Cytokine, activation marker, and chemokine receptor expression by individual CD4(+) memory T cells in rheumatoid arthritis synovium. Arthritis Res (2000) 2(5):415–23. doi: 10.1186/ar120 PMC17818 11056676

[93] Taylor PC Peters AM Paleolog E Chapman PT Elliott MJ McCloskey R. Reduction of chemokine levels and leukocyte traffic to joints by tumor necrosis factor alpha blockade in patients with rheumatoid arthritis. Arthritis Rheum (2000) 43(1):38–47. doi: 10.1002/1529-0131(200001)43:1<38::AID-ANR6>3.0.CO;2-L 10643698

[94] Garred P Madsen HO Petersen J Marquart H Hansen TM Freiesleben Sørensen S. CC chemokine receptor 5 polymorphism in rheumatoid arthritis. J Rheumatol (1998) 25(8):1462–5. 9712084

[95] Wang K Grivennikov SI Karin M. Implications of anti-cytokine therapy in colorectal cancer and autoimmune diseases. Ann Rheum Dis (2013) 72 Suppl 2:ii100–3. doi: 10.1136/annrheumdis-2012-202201 23253923

[96] Kol A Libby P. Molecular mediators of arterial inflammation: a role for microbial products? Am Heart J (1999) 138(5 Pt 2):S450–2. doi: 10.1016/S0002-8703(99)70273-5 10539846

[97] Nelken NA Coughlin SR Gordon D Wilcox JN. Monocyte chemoattractant protein-1 in human atheromatous plaques. J Clin Invest (1991) 88(4):1121–7. doi: 10.1172/JCI115411 PMC295565 1843454

[98] Ylä-Herttuala S Lipton BA Rosenfeld ME Särkioja T Yoshimura T Leonard EJ. Expression of monocyte chemoattractant protein 1 in macrophage-rich areas of human and rabbit atherosclerotic lesions. Proc Natl Acad Sci U.S.A. (1991) 88(12):5252–6. doi: 10.1073/pnas.88.12.5252 PMC51850 2052604

[99] Wilcox JN Nelken NA Coughlin SR Gordon D Schall TJ. Local expression of inflammatory cytokines in human atherosclerotic plaques. J Atheroscler Thromb (1994) 1 Suppl 1:S10–3. doi: 10.5551/jat1994.1.Supplemment1_S10 9222884

[100] Haley KJ Lilly CM Yang JH Feng Y Kennedy SP Turi TG. Overexpression of eotaxin and the CCR3 receptor in human atherosclerosis: using genomic technology to identify a potential novel pathway of vascular inflammation. Circulation (2000) 102(18):2185–9. doi: 10.1161/01.CIR.102.18.2185 11056090

[101] Fischman DL Leon MB Baim DS Schatz RA Savage MP Penn I. A randomized comparison of coronary-stent placement and balloon angioplasty in the treatment of coronary artery disease. Stent Restenosis Study Investigators N Engl J Med (1994) 331(8):496–501. doi: 10.1056/NEJM199408253310802 8041414

[102] Fattori R Piva T. Drug-eluting stents in vascular intervention. Lancet (2003) 361(9353):247–9. doi: 10.1016/S0140-6736(03)12275-1 12547552

[103] Richmond A Balentien E Thomas HG Flaggs G Barton DE Spiess J. Molecular characterization and chromosomal mapping of melanoma growth stimulatory activity, a growth factor structurally related to beta-thromboglobulin. EMBO J (1988) 7(7):2025–33. doi: 10.1002/j.1460-2075.1988.tb03042.x PMC454478 2970963

[104] Wang J Huang M Lee P Komanduri K Sharma S Chen G. Interleukin-8 inhibits non-small cell lung cancer proliferation: a possible role for regulation of tumor growth by autocrine and paracrine pathways. J Interferon Cytokine Res (1996) 16(1):53–60. doi: 10.1089/jir.1996.16.53 8640452

[105] Strieter RM Polverini PJ Kunkel SL Arenberg DA Burdick MD Kasper J. The functional role of the ELR motif in CXC chemokine-mediated angiogenesis. J Biol Chem (1995) 270(45):27348–57. doi: 10.1074/jbc.270.45.27348 7592998

[106] Tergaonkar V Correa R Ikawa M Verma IM. Distinct roles of IkappaB proteins in regulating constitutive NF-kappaB activity. Nat Cell Biol (2005) 7(9):921–3. doi: 10.1038/ncb1296 16136188

[107] Zhang Q Lenardo M Baltimore D. 30 years of NF-κB: A blossoming of relevance to human pathobiology. Cell (2017) 168:37–57. doi: 10.1016/j.cell.2016.12.012 28086098 PMC5268070

[108] Sun SC. The non-canonical NF-κB pathway in immunity and inflammation. Nat Rev Immunol (2017) 17(9):545–58. doi: 10.1038/nri.2017.52 PMC5753586 28580957

[109] Chen C Ai Q Wei Y. Hydroxytyrosol protects against cisplatin-induced nephrotoxicity *via* attenuating CKLF1 mediated inflammation, and inhibiting oxidative stress and apoptosis. Int Immunopharmacol (2021) 96:107805. doi: 10.1016/j.intimp.2021.107805 34162164

[110] Kyriakis JM Avruch J. Mammalian MAPK signal transduction pathways activated by stress and inflammation: a 10-year update. Physiol Rev (2012) 92(2):689–737. doi: 10.1152/physrev.00028.2011 22535895

[111] Kong LL Wang ZY Hu JF Yuan YH Han N Li H. Inhibition of chemokine-like factor 1 protects against focal cerebral ischemia through the promotion of energy metabolism and anti-apoptotic effect. Neurochem Int (2014) 76:91–8. doi: 10.1016/j.neuint.2014.07.004 25042180

[112] Li FF Zhou X Chu SF Chen NH. Inhibition of CKLF1 ameliorates hepatic ischemia-reperfusion injury *via* MAPK pathway. Cytokine (2021) 141:155429. doi: 10.1016/j.cyto.2021.155429 33578361

[113] Yu H Lin L Zhang Z Zhang H Hu H. Targeting NF-κB pathway for the therapy of diseases: mechanism and clinical study. Signal Transduct Target Ther (2020) 5(1):209. doi: 10.1038/s41392-020-00312-6 32958760 PMC7506548

[114] Macha MA Matta A Kaur J Chauhan SS Thakar A Shukla NK. Prognostic significance of nuclear pSTAT3 in oral cancer. Head Neck (2011) 33(4):482–9. doi: 10.1002/hed.21468 20652980

[115] Chen Y Wang J Wang X Liu X Li H Lv Q. STAT3, a poor survival predicator, is associated with lymph node metastasis from breast cancer. J Breast Cancer (2013) 16(1):40–9. doi: 10.4048/jbc.2013.16.1.40 PMC3625768 23593080

[116] Johnson DE O’Keefe RA Grandis JR. Targeting the IL-6/JAK/STAT3 signalling axis in cancer. Nat Rev Clin Oncol (2018) 15(4):234–48. doi: 10.1038/nrclinonc.2018.8 PMC5858971 29405201

[117] Paul J Soujon M Wengner AM Zitzmann-Kolbe S Sturz A Haike K. Simultaneous inhibition of PI3Kδ and PI3Kα induces ABC-DLBCL regression by blocking BCR-dependent and -independent activation of NF-κB and AKT. Cancer Cell (2017) 31(1):64–78. doi: 10.1016/j.ccell.2016.12.003 28073005

[118] Fruman DA Chiu H Hopkins BD Bagrodia S Cantley LC Abraham RT. The PI3K pathway in human disease. Cell (2017) 170(4):605–35. doi: 10.1016/j.cell.2017.07.029 PMC5726441 28802037

[119] Hoxhaj G Manning BD. The PI3K-AKT network at the interface of oncogenic signalling and cancer metabolism. Nat Rev Cancer (2020) 20(2):74–88. doi: 10.1038/s41568-019-0216-7 31686003 PMC7314312

[120] Chen L Monti S Juszczynski P Ouyang J Chapuy B Neuberg D. SYK inhibition modulates distinct PI3K/AKT- dependent survival pathways and cholesterol biosynthesis in diffuse large b cell lymphomas. Cancer Cell (2013) 23(6):826–38. doi: 10.1016/j.ccr.2013.05.002 PMC3700321 23764004

[121] Galasso JM Harrison JK Silverstein FS. Excitotoxic brain injury stimulates expression of the chemokine receptor CCR5 in neonatal rats. Am J Pathol (1998) 153(5):1631–40. doi: 10.1016/S0002-9440(10)65752-5 PMC1853404 9811356

[122] Simpson J Rezaie P Newcombe J Cuzner ML Male D Woodroofe MN. Expression of the beta-chemokine receptors CCR2, CCR3 and CCR5 in multiple sclerosis central nervous system tissue. J Neuroimmunol (2000) 108(1-2):192–200. doi: 10.1016/S0165-5728(00)00274-5 10900353

[123] Latz E Xiao TS and Stutz A. Activation and regulation of the inflammasomes. Nat Rev Immunol (2013) 13(6):397–411. doi: 10.1038/nri3452 23702978 PMC3807999

[124] Ai QD Chen C Chu S Zhang Z Luo Y Guan F. IMM-H004 therapy for permanent focal ischemic cerebral injury *via* CKLF1/CCR4-mediated NLRP3 inflammasome activation. Transl Res (2019) 212:36–53. doi: 10.1016/j.trsl.2019.05.007 31176667

[125] Yu H Pardoll D Jove R. STATs in cancer inflammation and immunity: a leading role for STAT3. Nat Rev Cancer (2009) 9(11):798–809. doi: 10.1038/nrc2734 19851315 PMC4856025

[126] Ibrahim SSA Huttunen KM. Orchestrated modulation of rheumatoid arthritis *via* crosstalking intracellular signaling pathways. Inflammopharmacology (2021) 29(4):965–74. doi: 10.1007/s10787-021-00800-3 33740220

[127] Johnson GL Lapadat R. Mitogen-activated protein kinase pathways mediated by ERK, JNK, and p38 protein kinases. Science (2002) 298(5600):1911–2. doi: 10.1126/science.1072682 12471242

[128] Manning AM Davis RJ. Targeting JNK for therapeutic benefit: from junk to gold? Nat Rev Drug Discovery (2003) 2(7):554–65. doi: 10.1038/nrd1132 12815381

[129] Bachstetter AD Xing B de Almeida L Dimayuga ER Watterson DM Van Eldik LJ. Microglial p38α MAPK is a key regulator of proinflammatory cytokine up-regulation induced by toll-like receptor (TLR) ligands or beta-amyloid (Aβ). J Neuroinflamm (2011) 8:79. doi: 10.1186/1742-2094-8-79 PMC3142505 21733175

[130] Xing B Bachstetter AD Van Eldik LJ. Microglial p38α MAPK is critical for LPS-induced neuron degeneration, through a mechanism involving TNFα. Mol Neurodegener (2011) 6:84. doi: 10.1186/1750-1326-6-84 22185458 PMC3292986

[131] Takeuchi O Akira S. Pattern recognition receptors and inflammation. Cell (2010) 140(6):805–20. doi: 10.1016/j.cell.2010.01.022 20303872

[132] Pease JE Horuk R. Chemokine receptor antagonists: Part 1. Expert Opin Ther Pat (2009) 19(1):39–58. doi: 10.1517/13543770802641346 19441897

[133] Li Z Hu J Sun M Ji H Chu S Liu G. Anti-inflammatory effect of IMMLG5521, a coumarin derivative, on sephadex-induced lung inflammation in rats. Int Immunopharmacol (2012) 14(2):145–9. doi: 10.1016/j.intimp.2012.06.004 22771447

[134] Song XY Hu JF Sun MN Li ZP Zhu ZX Song LK. IMM-H004, a novel coumarin derivative compound, attenuates the production of inflammatory mediatory mediators in lipopolysaccharide-activated BV2 microglia. Brain Res Bull (2014) 106:30–8. doi: 10.1016/j.brainresbull.2014.05.002 24878446

[135] Ai Q Chen C Chu S Luo Y Zhang Z Zhang S. IMM-H004 protects against cerebral ischemia injury and cardiopulmonary complications *via* CKLF1 mediated inflammation pathway in adult and aged rats. Int J Mol Sci (2019) 20(7). doi: 10.3390/ijms20071661 PMC6480569 30987181

[136] Viney JM Andrew DP Phillips RM Meiser A Patel P Lennartz-Walker M. Distinct conformations of the chemokine receptor CCR4 with implications for its targeting in allergy. J Immunol (2014) 192(7):3419–27. doi: 10.4049/jimmunol.1300232 PMC3965571 24563252

[137] Degirmenci U Wang M Hu J. Targeting aberrant RAS/RAF/MEK/ERK signaling for cancer therapy. Cells (2020) 9(1). doi: 10.3390/cells9010198 PMC7017232 31941155

[138] Pola R Flex A Gaetani E Proia AS Papaleo P Di Giorgio A. Monocyte chemoattractant protein-1 (MCP-1) gene polymorphism and risk of alzheimer’s disease in italians. Exp Gerontol (2004) 39(8):1249–52. doi: 10.1016/j.exger.2004.05.001 15288699

[139] Pham MH Bonello GB Castiblanco J Le T Sigala J He W. The rs1024611 regulatory region polymorphism is associated with CCL2 allelic expression imbalance. PloS One (2012) 7(11):e49498. doi: 10.1371/journal.pone.0049498 23166687 PMC3500309

[140] Pineda-Tenor D Berenguer J García-Álvarez M Guzmán-Fulgencio M Carrero A Aldámiz-Echevarria T. Single nucleotide polymorphisms of CXCL9-11 chemokines are associated with liver fibrosis in HIV/HCV-coinfected patients. J Acquir Immune Defic Syndr (2015) 68(4):386–95. doi: 10.1097/QAI.0000000000000491 25559603

[141] Ohmori Y Schreiber RD Hamilton TA. Synergy between interferon-gamma and tumor necrosis factor-alpha in transcriptional activation is mediated by cooperation between signal transducer and activator of transcription 1 and nuclear factor kappaB. J Biol Chem (1997) 272(23):14899–907. doi: 10.1074/jbc.272.23.14899 9169460

[142] Jin F Li Y Dixon JR Selvaraj S Ye Z Lee AY. A high-resolution map of the three-dimensional chromatin interactome in human cells. Nature (2013) 503(7475):290–4. doi: 10.1038/nature12644 PMC3838900 24141950

[143] Fok ET Davignon L Fanucchi S Mhlanga MM. The lncRNA connection between cellular metabolism and epigenetics in trained immunity. Front Immunol (2018) 9:3184. doi: 10.3389/fimmu.2018.03184 30761161 PMC6361822

[144] Fanucchi S Fok ET Dalla E Shibayama Y Börner K Chang EY. Immune genes are primed for robust transcription by proximal long noncoding RNAs located in nuclear compartments. Nat Genet (2019) 51(1):138–50. doi: 10.1038/s41588-018-0298-2 30531872

[145] Zhou X Zhang YN Li FF Zhang Z Cui LY He HY. Neuronal chemokine-like-factor 1 (CKLF1) up-regulation promotes M1 polarization of microglia in rat brain after stroke. Acta Pharmacol Sin (2022) 43(5):1217–30. doi: 10.1038/s41401-021-00746-w PMC9061752 34385606

[146] Guo S Wang H Yin Y. Microglia polarization from M1 to M2 in neurodegenerative diseases. Front Aging Neurosci (2022) 14:815347. doi: 10.3389/fnagi.2022.815347 35250543 PMC8888930

